# Association between prolactin increasing antipsychotic use and the risk of breast cancer: a retrospective observational cohort study in a United States Medicaid population

**DOI:** 10.3389/fonc.2024.1356640

**Published:** 2024-03-25

**Authors:** David M. Kern, Azza Shoaibi, David Shearer, Ute Richarz, Leslie Killion, R. Karl Knight

**Affiliations:** ^1^ Janssen Research & Development, LLC, Horsham, PA, United States; ^2^ Janssen Research & Development, LLC, Titusville, NJ, United States; ^3^ Janssen Research & Development, LLC, Zug, Switzerland

**Keywords:** antipsychotics, real-world evidence, prolactin, breast cancer, pharmacoepidemiology, comparative safety

## Abstract

**Introduction:**

Results of retrospective studies examining the relationship between prolactin increasing antipsychotics and incident breast cancer have been inconsistent. This study assessed the association between use of high prolactin increasing antipsychotics (HPD) and the incidence of breast cancer using best practices in pharmacoepidemiology.

**Methods:**

Using administrative claims data from the MarketScan Medicaid database, schizophrenia patients initiating antipsychotics were identified. Those initiating HPD were compared with new users of non/low prolactin increasing drugs (NPD). Two definitions of breast cancer, two at-risk periods, and two large-scale propensity score (PS) adjustment methods were used in separate analyses. PS models included all previously diagnosed conditions, medication use, demographics, and other available medical history. Negative control outcomes were used for empirical calibration.

**Results:**

Five analysis variants passed all diagnostics for sufficient statistical power and balance across all covariates. Four of the five variants used an intent-to-treat (ITT) approach. Between 4,256 and 6,341 patients were included in each group for the ITT analyses, and patients contributed approximately four years of follow-up time on average. There was no statistically significant association between exposure to HPD and risk of incident breast cancer in any analysis, and hazard ratios remained close to 1.0, ranging from 0.96 (95% confidence interval 0.62 - 1.48) to 1.28 (0.40 - 4.07).

**Discussion:**

Using multiple PS methods, outcome definitions and at-risk periods provided robust and consistent results which found no evidence of an association between use of HPD and risk of breast cancer.

## 1 Introduction

It is estimated that 287,500 women were newly diagnosed with breast cancer in the United States in 2022 (1). Approximately 1 in 8 women in the United States (US) will be diagnosed with the disease during their lifetime, making it the most common cancer in the country, and nearly 3% of women will die from breast cancer (1, 2).

The potential association between elevated prolactin levels and human breast cancer has been a topic of interest ever since studies in rodents demonstrated a causal relationship between prolactin and initiation and growth of tumors in the 1970s (3), and evidence collected in the subsequent decades has been well summarized (4). Large prospective cohort studies examining the association between prolactin levels and risk of breast cancers in humans have shown varied findings (5, 6). The possible link between prolactin levels and breast cancer has led to a concern regarding the use of antipsychotic drugs, including first generation antipsychotics and some second generation medications, because of the known effect of these agents to increase prolactin levels (7, 8). In 1978, the US Food and Drug Administration (FDA) was the first regulatory agency to require a class warning for all dopamine (D_2_) antagonists regarding hyperprolactinemia and potential risk to patients (9). However, the warning states that the available evidence is considered too limited to be conclusive.

Numerous observational studies have been conducted examining the relationship between antipsychotic use and breast cancer outcomes. In a dozen such studies that were reviewed, findings were mixed (10–21). Many studies found no evidence of association between exposure to prolactin increasing antipsychotics and breast cancer, while others, reported positive associations, including recent publications by Taipale et al. and Rahman et al. (10, 11). Many of these studies included significant limitations which could impact the validity of the findings. Such limitations include insufficient control for potential confounders, inappropriate comparators, the potential for confounding by indication, and study design and statistical modeling choices.

This study utilized a retrospective comparative cohort approach, aided by large scale propensity score modeling, to examine the association between use of prolactin increasing antipsychotics and the risk of incident breast cancer in patients diagnosed with schizophrenia. Specifically, we estimated the relative risk of a new diagnosis of breast cancer among new users of high prolactin increasing antipsychotic drugs (HPDs) compared with new users of non/low prolactin increasing drugs (NPDs).

## 2 Materials and methods

**Figure 1:**
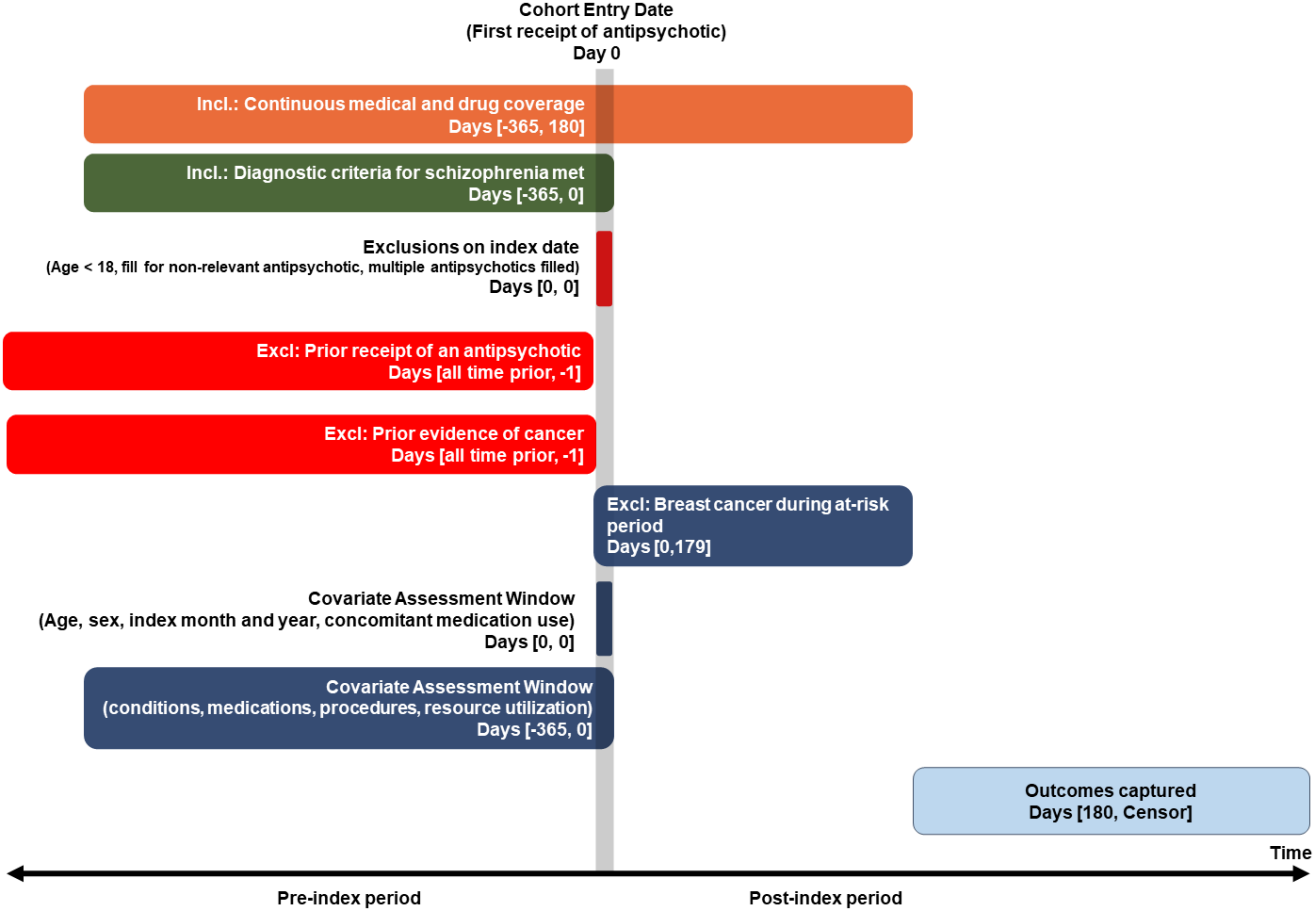
Retrospective comparative cohort study design requiring 180-day minimum exposure period (Primary, per protocol, analysis).

This study followed a retrospective new-user comparative cohort design detailed in the protocol found in the **Supplementary Material**. **Figure 1** illustrates the retrospective study design schematic. The study period started January 2006 and ended June 2021.

### 2.1 Data source

This retrospective longitudinal comparative cohort study used administrative health insurance claims data from the US. Five databases were considered for inclusion and included commercially insured individuals (Merative MarketScan^®^ Commercial Database, Optum^©^ Clinformatics^®^ Data Mart and IQVIA Adjudicated Health Plan Claims Data), Medicare eligible patients (Merative MarketScan^®^ Medicare Supplemental Database), and Medicaid patients (Merative MarketScan^®^ Multi-State Medicaid Database). Only the Medicaid database passed all pre-defined diagnostic criteria. The other databases contained too few patients meeting inclusion criteria, resulting in an inability to balance cohorts on baseline characteristics and insufficient power.

The MarketScan Medicaid database includes more than 33 million Medicaid enrollees from multiple states during 1 Jan 2006 through 30 Jun 2021. Data elements include outpatient pharmacy dispensing claims (coded with National Drug Codes) as well as inpatient and outpatient medical claims, which provide diagnosis codes (coded in ICD-9-CM or ICD-10-CM). All data were standardized to the Observational Health and Data Sciences and Informatics Observational Medical Outcomes Partnership Common Data Model version 5.3.

The use of the MarketScan databases was reviewed by the New England Institutional Review Board (IRB) and was determined to be exempt from broad IRB approval, as this research project did not involve human subjects research.

### 2.2 Study population(s)

All patients had a prescription fill for an antipsychotic medication categorized as either “high prolactin increasing”, “moderate prolactin increasing”, or “minimal/non-prolactin increasing”. The list of medications in each group can be found in the **Supplementary Material**. The first observed fill for an antipsychotic was considered the index date. Patients included women at least 18 years old on the index date, with at least 365 days of prior observation during which time there was a diagnosis of schizophrenia. Patients with prior exposure to an antipsychotic any time prior to the index date were excluded, with the exception of injectable therapies used for the acute management of psychotic symptoms. Those with more than one antipsychotic filled on the index date or with a fill for an antipsychotic typically used for treating conditions other than schizophrenia were excluded (see **Supplementary Materials** for codes). Patients with a diagnosis of cancer other than non-melanoma skin cancer any time prior to the index date, and those with presence of a mastectomy or lumpectomy any time prior to index, or a diagnosis of breast cancer during the first 180 days of post-index follow-up, were excluded.

### 2.3 Exposure

Two potential exposure cohorts were considered based on the index antipsychotic: patients initiating high prolactin increasing drugs (HPD) and those initiating moderate prolactin increasing drugs (MPD). A third cohort of patients exposed to non/low prolactin increasing drugs (NPD) was used as a comparator.

Assignment to the exposure or comparator group was determined on the index date, i.e., the date of the first observed fill for non-acute antipsychotic use. Exposure ended when a patient discontinued medication, defined as a gap in treatment within the exposure group of more than 30 days beyond the supply days of the last fill, an outcome occurred, or patient observation was censored. Censoring occurred due to being lost to follow-up, i.e., patient left the health plan and was not observed in the database, reaching end of database availability, or patient received an antipsychotic for a group other than the original group. Switching to an antipsychotic within the original assigned group was allowed and was considered as continuous use assuming the switch occurred before the criteria for discontinuation was met.

### 2.4 Outcome

The outcome of interest was incident, newly diagnosed, breast cancer. Two outcome definitions were implemented. The first was an algorithm created by Nattinger et al. (22), which was developed using Medicare claims data and validated against the gold-standard SEER classification. The Nattinger algorithm uses four steps to identify breast cancer cases, including a screening step, two steps aimed to confirm the case status, and a final step to remove prevalent cases. The steps include a combination of diagnosis codes, breast cancer related procedures, and surgical interventions. The original publication was from 2004 and did not include ICD-10-CM codes and many CPT codes that now exist. These codes were added where relevant.

The second definition (i.e., the “Rahman algorithm”) reflects methods most typically used in breast cancer research in claims data (10). This algorithm is less complex and easier to implement than the Nattinger algorithm; however, it has not been validated. The Rahman algorithm uses a combination of the presence of diagnosis codes, surgical treatments, and chemotherapies. The logic and codes used for both algorithms can be found in the **Supplementary Material**.

All definitions (exposure and outcomes) used in the study were implemented in the Medicaid database. Demographic and clinical profiles of identified individuals for each definition were characterized using the OHDSI CohortDiagnostics R package (23). The group level characterizations were reviewed to assess consistency with expectations.

### 2.5 Covariates

Patient characteristics were described for each treatment group, and all were considered for inclusion in the propensity score model. Variables captured included demographics, comorbid conditions, prior and concomitant medication use, prior procedures and measurements performed, and prior healthcare utilization. The complete list of covariates by domain can be found in the **Supplementary Material**. In all, more than 25,000 covariates were characterized and balanced between treatment groups.

### 2.6 Data analysis plan

#### 2.6.1 Time at risk

The at-risk period started 180 days following the index date. The six-month lag was required to make a reasonable inference that the exposure contributed to the incidence of cancer. While it is likely that a tumor may have been growing for years or decades prior to the first diagnosis, it is not feasible to require such extended observation periods. A hypothesized effect of prolactin on breast cancer is increased tumor growth not the formation of a tumor or initial cell mutation, thus long lag periods to detect *new* tumor formation may not be necessary (24).

Two different analyses were conducted, based on differing end date definitions of the at-risk period. An intent-to-treat (ITT) analysis considered the end of the risk period as the end of observation in the database (due to death, leaving the health plan, or reaching the end of the study period), regardless of continuous exposure to the index treatment group or exposure to the comparator. This analysis did not impose a minimum length of exposure to drug, but the at-risk period began on day 180. A per-protocol (‘on-treatment’) analysis was also conducted which considered the end date as the end of continuous exposure to the index medication class or presence of a censoring event as described in the “Exposure” section.

#### 2.6.2 Control for confounding

Propensity score matching and stratification techniques were used to control for observed potential confounding. The variables described in the “Covariates” section were considered for inclusion in the propensity score model used to predict treatment arm. A regression model using L1 regularization (LASSO regression), with the optimal hyperparameter to fit the model determined through 10-fold cross validation, was used to select the most relevant covariates for inclusion in the propensity score model.

Both PS 1:1 matching and PS stratification were considered. For matching, propensity scores were used to match the cohorts (using a caliper of 0.2 of the standardized logit score), in which a subset of the cohorts which were most similar to each other were retained. For stratification, to control for measured confounders, target and comparator patients were allocated into five strata defined by the distribution of the PS. Effects were estimated within strata then combined into a weighted average. Large-scale PS adjustment using five strata or 1:1 matching has been demonstrated to control the bias produced by measured confounders (25).

Covariate balance between the cohorts was assessed before and after the PS adjustment. Covariates were considered well balanced between cohorts if the standardized difference of means (SDM) was ≤0.10 (26). If sufficient overlap between propensity scores was not present and/or if matching/stratification the cohorts did not result in groups that are well balanced on all patient covariates listed previously, a decision was made to not proceed with the study due to an inability to make unbiased inferences regarding the relationship between exposure and outcome. See further detail in “Study diagnostics”.

#### 2.6.3 Outcome model specification

Cox proportional hazard models were used to estimate hazard ratios (HRs). The regression for the outcome models were conditioned on the PS match/strata with treatment as the sole explanatory variable. To adjust for potential unobserved confounding and residual bias, empirical calibration using negative controls was performed. Uncalibrated and calibrated results, including estimates and 95% confidence intervals, were calculated, but only calibrated results were used for inference.

#### 2.6.4 Analysis variants

Due to the different risk window end dates (per-protocol, intent-to-treat), outcome/breast cancer definition (Nattinger, Rahman), treatment/target groups (high prolactin-increasing, moderate prolactin-increasing), and propensity score methods (matching, stratification), there were 16 potential unique analysis variants (see **Supplementary Material**).

#### 2.6.5 Study diagnostics

The study was subject to a set of pre-determined standardized diagnostics. Each diagnostic had a failure threshold, and failure of any single diagnostic resulted in that analysis not being conducted. Passing study diagnostics included a minimum detectable relative risk (MDRR) of 10 with α=0.05 and β=0.20, maximum attrition from propensity score matching or stratification of 50%, at least 10% of the population in statistical equipoise (27), and all covariates being well balanced (SDM ≤0.10) (26).

While 16 analysis variants were considered, most did not pass diagnostics, typically due to insufficient sample sizes and few outcomes leading to MDRRs larger than 10.0 and/or insufficiently balanced covariates. Ultimately, a single database and single exposure cohort were used, MarketScan Medicaid and the high prolactin-increasing antipsychotic group, respectively. There were four analysis variants which used the ITT approach. Just one variant of the on-treatment time at risk passed diagnostics.

## 3 Results

### 3.1 Patient characterization

Patients were well balanced on demographics and medical history after propensity score methods were implemented. **Table 1** illustrates results from PS matching and similar balance was achieved in the PS stratification adjustment. The age of women ranged from 18 to greater than 85 years, with a mean age of 48 years. Patients were racially diverse with 45% who were black and 41% white. Common comorbid conditions included depression, anxiety, bipolar disorder, diabetes, hyperlipidemia, hypertension, obesity and osteoarthritis. Use of antidepressants, antiepileptics, anxiolytics, and hypnotics/sedatives were commonly observed at baseline.

**Table 1:** Cohorts’ characteristics of new users of high prolactin-increasing antipsychotic (target) and new users of non-prolactin increasing antipsychotic (comparator) users, we report the proportion of based-line characteristics and the standardized mean difference (SMD) before and after matching.

Characteristic	Before PS matching			After PS matching		
	Target %	Comparator %	SMD	Target %	Comparator %	SMD
Age group						
18 - 19	2.4	2.2	0.01	2.1	2.0	0.00
20 - 24	6.5	7.4	-0.04	6.5	6.6	0.00
25 - 29	9.2	10.2	-0.03	9.3	9.1	0.01
30 - 34	8.7	11.2	-0.08	9.1	9.9	-0.03
35 - 39	8.4	10.4	-0.07	8.8	10.1	-0.04
40 - 44	9.1	9.8	-0.02	9.7	9.8	0.00
45 - 49	11.5	11.2	0.01	11.6	11.4	0.00
50 - 54	12.1	11.5	0.02	12.8	12.2	0.02
55 - 59	11.6	10.4	0.04	11.4	11.1	0.01
60 - 64	9.0	7.3	0.06	8.6	8.0	0.02
65 - 69	4.3	3.1	0.06	3.8	3.7	0.00
70 - 74	2.7	1.9	0.05	2.6	2.3	0.02
75 - 79	2.0	1.5	0.04	1.7	1.6	0.00
80 - 84	1.4	1.1	0.03	1.2	1.4	-0.01
85 - 89	1.0	0.7	0.03	0.9	0.9	0.01
Race						
Black or African American	50.1	40.5	0.19	44.6	45.9	-0.03
White	36.6	44.9	-0.17	41.8	41.0	0.01
Ethnicity: Hispanic or Latino	1.5	1.9	-0.03	1.6	1.3	0.02
Medical history: Mental health conditions						
Anxiety disorder	28.9	41.8	-0.27	35.0	32.4	0.06
Attention deficit hyperactivity disorder	2.9	4.6	-0.09	3.4	3.3	0.01
Bipolar disorder	34.5	46.2	-0.41	40.5	38.8	0.03
Dementia	7.8	6.3	0.06	7.4	7.1	0.01
Depressive disorder	47.3	58.2	-0.22	53.5	52.0	0.03
Substance use disorder	16.8	20.5	-0.10	18.4	17.4	0.03
Medical history: Other conditions						
Chronic obstructive lung disease	12.7	15.7	-0.09	14.8	14.1	0.02
Diabetes mellitus	25.2	24.4	0.02	26.0	25.0	0.02
Hyperlipidemia	25.7	26.2	-0.01	27.1	26.2	0.02
Hypertensive disorder	46.0	45.9	0.00	46.9	46.1	0.02
Obesity	18.8	21.6	-0.07	21.0	20.3	0.02
Osteoarthritis	18.9	23.5	-0.11	21.8	21.2	0.01
Prior use of prolactin increasing medication						
Metoclopramide	3.5	5.5	-0.10	4.5	4.3	0.01
Clomipramine	0.1	0.1	-0.01	0.1	0.2	-0.01
Fenfluramine	0.0	0.0	NA	0.0	0.0	NA
Cimetidine	0.0	0.0	-0.00	0.0	0.0	-0.02
Methyldopa	0.1	0.1	-0.00	0.1	0.0	0.01
Other common prior medication use						
Antidepressants	47.0	58.3	-0.23	53.5	51.7	0.04
Antiepileptics	30.7	40.7	-0.21	36.0	35.2	0.02
Anxiolytics	30.8	39.9	-0.19	35.6	34.0	0.03
Hypnotics and sedatives	46.0	57.6	-0.23	52.9	50.9	0.04
Opioids	26.6	37.5	-0.23	33.0	30.1	0.06
Psycholeptics	37.4	48.3	-0.22	43.3	41.9	0.03

SMD, standardized mean difference; PS, propensity score. Less extreme SMD through 1:1 matching suggests improved balance between patient cohorts through propensity score adjustment.

NA, not applicable.

**Table 2** illustrates patient cohort size, duration of follow-up, breast cancer cases count, and minimum detectible risk ratio for each group in each PS-adjustment analysis. The analysis with the largest sample size included more than 12,000 patients and included 80 outcomes of incident breast cancer. In the four intent-to-treat analyses there were between 4,256 and 6,341 patients included in each group, and patients contributed approximately four years of follow-up time on average. Additional data on the time-at-risk distributions for each analysis variant can be found in the **Supplementary Material**.

**Table 2:** Number of patients and breast cancer events for high prolactin-increasing antipsychotic users and non-prolactin-increasing antipsychotic users, we report population size, total exposure time, outcome events and minimal detectable risk ratio (MDRR) for the PS 1-1 matching analysis.

At-risk period and outcome	N patients		Follow-up time (total patient-years)		Breast cancer cases		MDRR	PS adjustment
	HPD	NPD	HPD	NPD	HPD	NPD		
Intent-to-treat								
Nattinger definition	4,256	4,256	17,157	17,621	28	28	2.11	1:1 matching
Nattinger definition	6,341	5,868	26,495	22,964	47	33	1.87	stratification
Rahman definition	4,256	4,256	17,157	17,621	36	34	1.93	1:1 matching
Rahman definition	6,341	5,867	26,480	22,940	55	41	1.77	stratification
On-treatment								
Rahman definition	2,687	2,506	4,008	3,697	10	5	4.25	stratification

HPD, high prolactin-increasing drugs; MDRR, minimal detectable relative risk; NPD, non-prolactin-increasing drugs; PS, propensity score.

### 3.2 Breast cancer risk

**Table 3** reports the calibrated hazard ratio estimating the risk of breast cancer among new users of HPD compared to NPD new users. There was no statistically significant association between the exposure to HPD use and risk of incident breast cancer in any of the analyses, and HRs remained close to the null effect of 1.0, ranging from 0.96 (95% confidence interval 0.62-1.48) to 1.28 (0.40 – 4.07) across different analysis variants. Results were largely consistent between the two outcome definitions and the two PS adjustment methods.

**Table 3:** Relative risk of breast cancer for high prolactin-increasing antipsychotic users, compared to non-prolactin-increasing antipsychotic users, we report calibrated hazard ratios (HRs) and their 95% confidence intervals (CIs) and p-value (P), with propensity score (PS) stratification or matching.

	PS-matched		PS-stratified	
	*HR (95% CI)	P-value	*HR (95% CI)	P-value
Intent-to-treat				
Nattinger definition	1.05 (0.62-1.78)	0.86	1.00 (0.62-1.60)	0.92
Rahman definition	1.11 (0.69-1.79)	0.66	0.96 (0.62-1.48)	0.85
On-treatment				
Rahman definition	NA	NA	1.28 (0.40-4.07)	0.68

PS, propensity score; HR, hazard ratio; NA, not applicable (analysis variant did not pass study diagnostics).

*Calibrated hazard ratio using distribution of negative controls.

**Figure 2:**
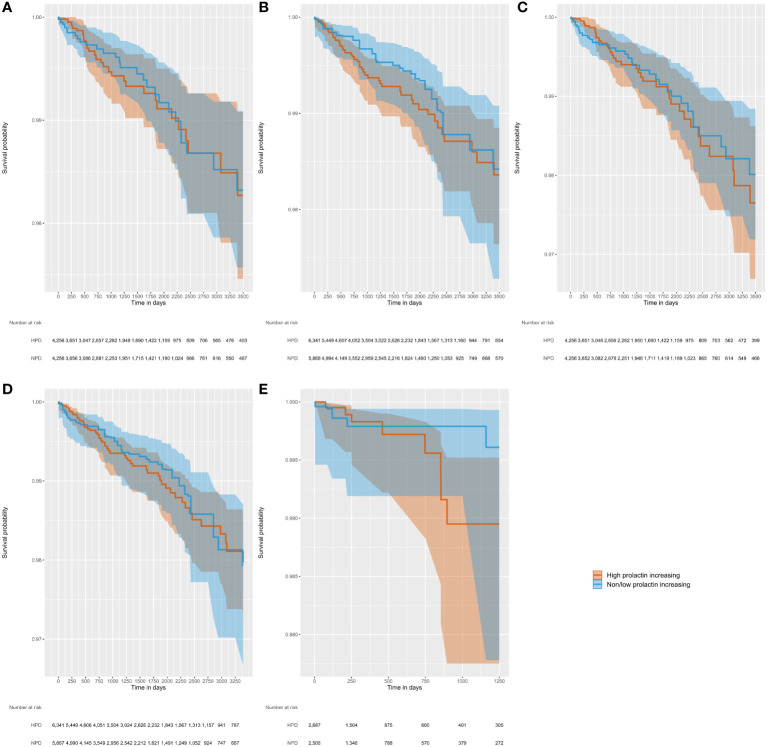
Kaplan-Meier figures, with shaded 95% confidence intervals, illustrating time to incident breast cancer diagnosis in five separate analysis variants varying by outcome definition, at-risk period, and PS method. **(A)** Outcome, Nattinger; at-risk period, ITT; PS method, matching; **(B)** Outcome, Nattinger; at-risk period, ITT; PS method, stratification; **(C)** Outcome, Rahman; at-risk period, ITT; PS method, matching; **(D)** Outcome, Rahman; at-risk period, ITT; PS method, stratification; **(E)** Outcome, Rahman; at-risk period, on treatment; PS method, stratification.

**Figure 2** displays the Kaplan Meier curves by analysis variant. The Kaplan Meier plot shows the survival as a function of time, with minimal separation between the two step functions, consistent with the results reported from the Cox models indicating no significant difference in the risk of breast cancer between the two exposure groups. The plots reflect propensity score adjusted survival estimates. The target curve (HPD users) shows the actual observed survival. The comparator curve (NPD users) applies reweighting to approximate the counterfactual of what the target survival would look like had the target cohort been exposed to the comparator instead.

## 4 Discussion

### 4.1 Key findings

This study assessed the association between high prolactin increasing antipsychotics versus non/low prolactin increasing antipsychotics and incident cases of breast cancer and found no evidence of association between the exposure and outcome. The finding was robust across two different outcome definitions, for varying times at risk and using different propensity score techniques for controlling for confounding. While multiple databases were considered for inclusion, just one, MarketScan Medicaid, passed all diagnostics required to perform the analysis. The inability to perform the analysis in other databases was due to a small number of exposures resulting in an inability to adjust for imbalances of covariates between cohorts and/or a small number of observed cases resulting in a lack of statistical power.

### 4.2 Strengths

This study followed best practices of pharmacoepidemiology and used advanced epidemiologic methods to reduce potential impacts of observed and unobserved confounding, and resulting biases, as much as possible. The methods used in this design improve upon recent previous research examining the relationship between antipsychotic use and breast cancer. The study by Taipale et al. reported increased odds of high prolactin antipsychotic use in breast cancer cases (11). However, this study used a nested case-control design, which has been shown to be prone to producing biased estimates and has led to a call to avoid their use when using retrospective data for which alternative designs are feasible and less biased, especially in the absence of negative control adjustment (28). In a recent publication by Rahman et al, the authors used a comparative cohort design to assess risk of breast cancer in antipsychotic users, but the choice of comparator, lithium or anticonvulsants, is susceptible to confounding by indication (10). Patients who received antipsychotics were likely much different, and in ways that could not be adjusted for, than those who received these other therapies. Prior to these recently published studies, others have examined the association between prolactin increasing antipsychotics and breast cancer risk with mixed findings in both case-control and retrospective cohort designs (15, 18, 19, 21).

This study used multiple techniques to reduce the amount of bias present. Two different algorithms were used for defining breast cancer, one which was more specific (Nattinger) and one that was more sensitive (Rahman), and results were consistent for both. The new-user design was able to capture events following treatment exposures while avoiding confounding from previous treatment effects, known as a prevalent user bias. New use allows for a clear exposure index date designation and a baseline period for which patient characteristics can be measured and balanced between groups. Using large-scale propensity score adjustment resulted in the balance of a large number (i.e., >25,000) of baseline potential confounders, minimizing the potential for confounding due to observable characteristics. A delayed time-at-risk start date, 180 days following initiation of antipsychotic therapy, reduced the potential for protopathic bias in which cases of breast cancer occurred prior to the index date but were not diagnosed until soon after. This decision also improved causal inference by including only long exposure and observation periods. Using negative control outcomes allowed for the estimation of residual bias inherent to the study design and data and provided the ability to calibrate study results to account for this residual bias.

The MarketScan Medicaid data contains a much smaller number of patients than the MarketScan Commercial and Clinformatics databases which include data from large private insurers. However, most of the identifiable schizophrenia patients were found in the Medicaid data, making it most fit for purpose and the most representative of the underlying schizophrenia population, though not necessarily representative of the US population as a whole.

### 4.3 Limitations

The confidence intervals of the hazard ratios are wide due to relatively few cases and do not rule out the possibility of small causal effects. However, all ITT analyses were powered to detect true hazard ratios of roughly 2.0 with 80% power, and all observed effect estimates were close to the null effect of 1.0.

The outcome of breast cancer relies on algorithms using administrative claims data, which, while shown to have high validity, are not perfect in terms of sensitivity or specificity. The presence of a diagnosis code signals a stage at which a tumor growth has led to seeking and receiving healthcare to address the cancer. Initial cell mutations and early tumor growth may have begun years prior to a diagnosis code being observed. However, *in vitro* and *in vivo* studies support that prolactin is involved in processes related to late-stage carcinogenic effects of breast cancer, including increasing cell proliferation and reducing apoptosis. Therefore, prolactin levels may only be important after a preclinical lesion has developed. The claims data do not have information on biomarkers, cancer grade, tumor staging, or other clinical measures for the outcome. Further, the claims data also lack detail on serum prolactin levels which are the hypothesized mediator between HPD use and potential risk of breast cancer. Thus, it is unclear whether the lack of an association is due to lack of a significant increase in prolactin levels, or if prolactin levels were increased but did not result in an increased risk of incident breast cancer.

While two different at-risk periods were considered, one which used an ITT approach and a second which considered only time exposed to treatment, the ITT approach accounted for four of the five analyses conducted. ITT is arguably the more appropriate study design choice for the outcome. Given the chronic nature of breast cancer and potentially long lead times from exposure to formation of cancer and finally diagnosis, the requirement that patients must be exposed to drug while experiencing the incident diagnosis of breast cancer is likely unnecessary and too conservative leading to substantial loss in statistical power. However, the mean follow-up in this study was approximately four years which may not be sufficient to capture an increased risk of breast cancer if truly present.

Causality between drug exposure and any given event cannot be drawn for individual cases. Socioeconomic variables (such as education and income), behavioral variables (such as diet, exercise, tobacco, and other drug use) were not available or may not be completely captured from these databases. Potential residual confounding may occur due to incomplete capture of other breast cancer risk factors (genetic mutations, family history, breast density, parity, menopause status etc.). Adjustment by propensity score may not completely remove confounding bias, specifically bias due to unobserved variables. However, it has been demonstrated that large scale propensity score adjustment can indirectly control unobserved variables (29). For example, while smoking is not fully captured in many data sources, adjusting for all observed health conditions and health utility variables that may be associated with smoking is likely to indirectly control for smoking.

### 4.4 Conclusions

A definitive association between the prolactin induced mammary tumors in rodents and the development or promotion of breast cancer in female patients with schizophrenia exposed to antipsychotics continues to be an area of debate. Both *in vitro* studies and epidemiologic studies have delivered conflicting results suggesting that if prolactin has any role it is limited. This retrospective cohort study adds to our knowledge by finding no association between prolactin raising antipsychotic use and breast cancer in women diagnosed with schizophrenia. Data available do not warrant a broad change in the approach to the treatment of women with schizophrenia with antipsychotics.

## Data availability statement

The data analyzed in this study is subject to the following licenses/restrictions: Data were licensed through a third party. Requests to access these datasets should be directed to Dave Kern, dkern2@its.jnj.com.

## Ethics statement

Ethical approval was not required for the study involving humans in accordance with the local legislation and institutional requirements. Written informed consent to participate in this study was not required from the participants or the participants’ legal guardians/next of kin in accordance with the national legislation and the institutional requirements.

## Author contributions

DK: Conceptualization, Data curation, Formal analysis, Investigation, Methodology, Project administration, Supervision, Writing – original draft, Writing – review & editing. AS: Formal analysis, Investigation, Methodology, Visualization, Writing – original draft, Writing – review & editing. DS: Conceptualization, Supervision, Writing – review & editing. UR: Methodology, Supervision, Writing – review & editing. LK: Conceptualization, Supervision, Writing – review & editing. RK: Conceptualization, Investigation, Methodology, Supervision, Writing – review & editing.
