# Supplementary material for: Association between prolactin increasing antipsychotic use and the risk of breast cancer: a retrospective observational cohort study in a United States Medicaid population
Source: Front Oncol. 2024 Mar 25;14:1356640. doi: 10.3389/fonc.2024.1356640 (PMC11003262; doi:10.3389/fonc.2024.1356640)
Supplement: Supplementary file 1 [file DataSheet_1.pdf]

Contents

1. Exposure definitions 2
2. Outcome definitions 5
    2.1. Algorithm #1: Nattinger Algorithm 5
    2.2. Algorithm #2: Rahman Algorithm 7
3. Analysis variants 10
4. Time at risk Distributions 11

1. EXPOSURE DEFINITIONS

Table 1. Schizophrenia codes

Code	Name	Vocabulary
F20.0	Paranoid schizophrenia	ICD10CM
F20.1	Disorganized schizophrenia	ICD10CM
F20.2	Catatonic schizophrenia	ICD10CM
F20.3	Undifferentiated schizophrenia	ICD10CM
F20.5	Residual schizophrenia	ICD10CM
F20.89	Other schizophrenia	ICD10CM
F20.9	Schizophrenia, unspecified	ICD10CM
295.00	Simple type schizophrenia, unspecified	ICD9CM
295.01	Simple type schizophrenia, subchronic	ICD9CM
295.02	Simple type schizophrenia, chronic	ICD9CM
295.03	Simple type schizophrenia, subchronic with acute exacerbation	ICD9CM
295.04	Simple type schizophrenia, chronic with acute exacerbation	ICD9CM
295.05	Simple type schizophrenia, in remission	ICD9CM
295.10	Disorganized type schizophrenia, unspecified	ICD9CM
295.11	Disorganized type schizophrenia, subchronic	ICD9CM
295.12	Disorganized type schizophrenia, chronic	ICD9CM
295.13	Disorganized type schizophrenia, subchronic with acute exacerbation	ICD9CM
295.14	Disorganized type schizophrenia, chronic with acute exacerbation	ICD9CM
295.15	Disorganized type schizophrenia, in remission	ICD9CM
295.20	Catatonic type schizophrenia, unspecified	ICD9CM
295.21	Catatonic type schizophrenia, subchronic	ICD9CM
295.22	Catatonic type schizophrenia, chronic	ICD9CM
295.23	Catatonic type schizophrenia, subchronic with acute exacerbation	ICD9CM
295.24	Catatonic type schizophrenia, chronic with acute exacerbation	ICD9CM
295.25	Catatonic type schizophrenia, in remission	ICD9CM
295.30	Paranoid type schizophrenia, unspecified	ICD9CM
295.31	Paranoid type schizophrenia, subchronic	ICD9CM
295.32	Paranoid type schizophrenia, chronic	ICD9CM
295.33	Paranoid type schizophrenia, subchronic with acute exacerbation	ICD9CM
295.34	Paranoid type schizophrenia, chronic with acute exacerbation	ICD9CM
295.35	Paranoid type schizophrenia, in remission	ICD9CM
295.50	Latent schizophrenia, unspecified	ICD9CM
295.51	Latent schizophrenia, subchronic	ICD9CM
295.52	Latent schizophrenia, chronic	ICD9CM
295.53	Latent schizophrenia, subchronic with acute exacerbation	ICD9CM
295.54	Latent schizophrenia, chronic with acute exacerbation	ICD9CM
295.55	Latent schizophrenia, in remission	ICD9CM
295.60	Schizophrenic disorders, residual type, unspecified	ICD9CM

295.61	Schizophrenic disorders, residual type, subchronic	ICD9CM
295.62	Schizophrenic disorders, residual type, chronic	ICD9CM
295.63	Schizophrenic disorders, residual type, subchronic with acute exacerbation	ICD9CM
295.64	Schizophrenic disorders, residual type, chronic with acute exacerbation	ICD9CM
295.65	Schizophrenic disorders, residual type, in remission	ICD9CM
295.75	Schizoaffective disorder, in remission	ICD9CM
295.80	Other specified types of schizophrenia, unspecified	ICD9CM
295.81	Other specified types of schizophrenia, subchronic	ICD9CM
295.82	Other specified types of schizophrenia, chronic	ICD9CM
295.83	Other specified types of schizophrenia, subchronic with acute exacerbation	ICD9CM
295.84	Other specified types of schizophrenia, chronic with acute exacerbation	ICD9CM
295.85	Other specified types of schizophrenia, in remission	ICD9CM
295.90	Unspecified schizophrenia, unspecified	ICD9CM
295.91	Unspecified schizophrenia, subchronic	ICD9CM
295.92	Unspecified schizophrenia, chronic	ICD9CM
295.93	Unspecified schizophrenia, subchronic with acute exacerbation	ICD9CM
295.94	Unspecified schizophrenia, chronic with acute exacerbation	ICD9CM
295.95	Unspecified schizophrenia, in remission	ICD9CM

Table 3. List of antipsychotics and their associated exposure group

Antipsychotic	Exposure group
aripiprazole	Non/low
asenapine	Non/low
brexpiprazole	Non/low
clozapine	Non/low
lumateperone	Non/low
quetiapine	Non/low
ziprasidone	Non/low
iloperidone	Moderate
lurasidone	Moderate
olanzapine	Moderate
acetophenazine	High
chlorpromazine	High
chlorprothixene	High
fluphenazine	High
haloperidol	High
loxapine	High
molindone	High
paliperidone	High
perphenazine	High
risperidone	High
thioridazine	High
thiothixene	High
trifluoperazine	High
droperidol	Other
pimozide	Other
prochlorperazine	Other
promazine	Other

"Other" antipsychotics are those used to primarily treat conditions other than schizophrenia and were used to establish clean period of no prior antipsychotic use but were not used for any of the exposure groups

2. OUTCOME DEFINITIONS

2.1. Algorithm #1: Nattinger Algorithm

The Nattinger algorithm uses 4 steps to identify breast cancer cases. All codes used to identify each of the items can be found in the table below. The original publication was from 2004 and did not include ICD-10-CM codes and many CPT codes that now exist. These codes have been added where relevant.

Step 1. Referred to as the "screen," requires that a potential case have both a breast cancer diagnosis code and a breast cancer procedure code (not necessarily on the same claim) within 365 days. Only subjects satisfying this screening step are retained for further consideration. The date of the earliest observed breast cancer diagnosis is the potential event date for the outcome.

Step 2. Directly includes subjects with a high likelihood of being a case. To be classified as a case based on this step, the subject must meet both of the following criteria:

- [A mastectomy claim] or [a lumpectomy or partial mastectomy claim followed by at least one outpatient or provider claim for radiotherapy with a breast cancer diagnosis].

- At least two outpatient or provider claims on different dates, within 365 days of each other, containing breast cancer.

Subjects who pass step 2 are classified as possible incident cases and proceed to step 4. Subjects who are not classified as cases at step 2 go to step 3.

Step 3. This step of the algorithm applies to all potential cases that passed the screen (step 1) but were not directly included at step 2. In practice, this step differentiates primary breast cancer cases from women undergoing lumpectomy or partial mastectomy for benign disease or for another cancer that had metastasized to the breast. Four different variables are needed for Step 3:

- Surgery. This variable is positive (i.e., set to a value of 1) if one or more lumpectomy, partial mastectomy, or mastectomy codes are found. Otherwise, the variable is negative (set to a value of zero).

- Single Claim. This variable is positive (i.e., set to a value of 1) if a woman with lumpectomy or partial mastectomy claim had only one month in which a claim contained a primary breast cancer or a breast carcinoma-in-situ diagnosis (i.e., there do not exist two claims for breast cancer that occur between 30 and 365 days of each other). Otherwise, this variable is negative (i.e., set to 0).

- Other Cancer. This variable is positive (i.e., set to 1) if an "other cancer" code is found in one or more claims any time prior or within 30 days following the breast cancer index date. Otherwise, this variable is set to 0.

- Secondary Cancer to Breast. This variable is positive (i.e., set to 1) if a code for secondary cancer to breast is found in one or more outpatient or provider claims any time prior or within 30 days following the breast cancer index date. Otherwise, this variable is set to 0.

Once the values of the four variables have been determined, subjects can be ruled in if they have one of three combinations of the variables. These combinations are:

(1) "Surgery" = 1 and the other three variables = 0, (i.e., the patient has surgery, two breast cancer claims between 30 and 365 days of each other, no other cancer diagnosis within 365 days, and no diagnosis of secondary cancer), or

(2) "Surgery" = 1, "other cancer" = 1, and the other two variables = 0, (i.e., patient has surgery, two breast cancer claims between 30 and 365 days of each other, no diagnosis of secondary cancer, but with claims for other cancer), or

(3) "Surgery" = 1, "secondary cancer to breast" = 1, and the other two variables = 0 (i.e., patient has surgery, two breast cancer claims between 30 and 365 days of each other, no claims for other cancer, but with claims for secondary cancer to breast).

In summary, patients must have a claim for a breast cancer related surgery and at least two diagnoses between 30 and 365 days of each other and without evidence of both "other cancer" and "secondary cancer to breast", though having one, and only one, of those is allowed. With all other combinations, the subject is declared not to be a breast cancer case (i.e., patient does not have a claim for surgery, does not have at least two claims with a breast cancer diagnosis within 30 to 365 days of each other, or has claims with diagnoses for other types of cancer and cancer secondary to breast).

Step 4. This step of the algorithm is the step to remove prevalent breast cancer cases. This step uses up to three prior years of claims of subjects classified as a case in step 2 or step 3. Such subjects are removed if they have a claim in prior years that was either positive for step 1 (the screening step) of the algorithm, or a diagnosis of prior history of breast cancer.

Table 4. Codes used for Nattinger algorithm

Diagnosis or Procedure	Diagnosis codes (ICD-9-CM/ ICD-10-CM)	Procedure codes (CPT/HCPCS and ICD-9 Procedure, ICD-10 PCS)
Breast cancer **	174.0–174.9, 175.0-175.9<br>All of C50*, includes: C50.011-C50.119, C50.111-C50.119, C50.211-C50.219, C50.311-C50.319, C50.411-C50.419, C50.511-C50.519, c50.6-c50.629	
Carcinoma-in-situ (breast) **	233.0<br>D05.10-D05.92	
Mastectomy †,§		ICD9: 85.33-85.36, 85.41-85.48<br>ICD10-CM: 0HTT0ZZ, 0HTU0ZZ, 0HTV0ZZ<br>CPT: 19180, 19182, 19200, 19220, 19240, 19303-19307
Lumpectomy and partial mastectomy †,§		ICD9: 85.20-85.23<br>ICD10-CM, 0HBT0ZZ, 0HBT7ZZ, 0HBT8ZZ, 0HBU0ZZ, 0HBU7ZZ, 0HBU8ZZ, 0HBV0ZZ, 0HBV7ZZ, 0HBV8ZZ, 0HTWXZZ, 0HTXXZZ, 0HBT3ZZ, 0HBU3ZZ, 0HBV3ZZ, 0HBW3ZZ, 0HBX3ZZ, 0HBW0ZZ, 0HBW7ZZ, 0HBW8ZZ, 0HBWXZZ, 0HBX0ZZ, 0HBX7ZZ, 0HBX8ZZ, 0HBXXZZ<br>CPT: 19120, 19125, 19126, 19160, 19162, 19301, 19302

Biopsy †		ICD9: 85.11, 85.12<br>ICD10-CM: 0HBT3ZX, , 0HBU3ZX, , 0HBV3ZX, , 0HBW3ZX, , 0HBX3ZX, , 0HBW0ZX, , 0HBW7ZX, 0HBW8ZX, , 0HBWXZX, , 0HBX0ZX, , 0HBX7ZX, 0HBX8ZX, , 0HBXXZX, , 0HBX8ZX, 0HBX7ZX, 0HBX3ZX,<br>0HBX0ZX, 0HBWXZX, 0HBW8ZX, 0HBW7ZX, 0HBW3ZX, 0HBW0ZX<br>CPT: 19081, 19082, 19083, 19084, 19085, 19086, 19100, 19101, 19102, 19103, 19125, 19126, 77031
Lymph node dissection associated with breast cancer (thorax, axillary, mammary) †,§		ICD9: 40.22, 40.23, 40.3, 40.51<br>ICD10-CM: 07B5*, 07B6*, 07B7*, 07B8*, 07B9*,07T50ZZ, 07T54ZZ,<br>07T60ZZ, 07T64ZZ<br><br>CPT: 38525, 38530, 38542, 38740, 38745, 38746
Secondary cancer to breast ^	198.2, 198.81, C79.2, C79.81	
Other cancer ^	140.0-208.91 (excluding 174.0-175.9, 196.0-196.9, 198.2, 198.81, 199.2), 230-239.9 (excluding 233.0, 238.3, 239.3)<br>C00-C80 (excluding C50.*, C77.*, C79.2, C79.81), D00-D09 (excluding D05.10-D05.92), D37-D48 (excluding D48.60, D48.61, D48.62,), D49 (excluding D49.3)	
History of breast cancer #	V10.3, Z85.3	
Tumor in breast of uncertain nature	238.3, 239.3<br>D48.60, D48.61, D48.62, D49.3	
Radiation therapy +	92.20–92.29, 92.30-92.39, 92.41	ICD9-CM procedure codes: 92.20–92.29, 92.30-92.39, 92.41<br>ICD10 PCS codes: DM0*, DM1*, DM2*, DMY*<br>CPT/HCPCS codes:<br>77371-77387, G6001, G6002, G6015-G6017 (stereoactic radiation),<br>77399-77417, G6003 - G6014 (radiation treatment),<br>77423-77425 (neutron beam treatment),<br>77427-77499 (radiation treatment management),<br>77520-77525 (proton beam radiation),<br>77600-77615 (radiation hyperthermia treatment),<br>77620 (clinical intracitary radiation hyperthermia treatment),<br>77750-77799 (clinical brachytherapy radiation treatment)<br>G6003 - G6014

** Diagnoses for Step 1
† Procedures for Step 1
§ Procedures for ‘‘Surgery’’ variable in Step 3
^ other variables used in Step 3
# Variable used in Step 4
+ Variable used in Step 2

2.2. Algorithm #2: Rahman Algorithm

Observational studies using administrative claims data typically require two claims with a diagnosis of breast cancer plus other criteria such as chemotherapy, mastectomy, or lumpectomy. A recent study published by Rahman et al [10] examined the association between prolactin-elevating antipsychotic drugs and used an algorithm less complicated than the one detailed above, but not overly simplistic as to severely limit the validity of the classification. All codes used in the algorithm can be found in the Table below.

The algorithm is as follows:

- **Criteria 1, automatic case**: Breast cancer is identified by the ICD-9/10-CM codes for breast cancer (invasive or *in situ*) on a claim with a CPT-4 procedure code for surgical pathology microscopic examination, indicating pathologic verification.

- **Criteria 2**: If criteria 1 is not met, the following two stipulations must be met:

    - a diagnosis of breast cancer on an inpatient facility claim or on at least 2 provider/outpatient claims separated by 30 to 180 days.

    - Evidence of surgical treatment (mastectomy or breast-conserving surgery within 1 month before through 6 months after first breast cancer diagnosis) or chemotherapy (within 6 months after first breast cancer diagnosis, chemotherapy administration coded for invasive breast cancer)

Table 5. Codes used for Rahman algorithm

Category	ICD-9-CM/ICD-10 Diagnosis Codes	ICD-9-CM/ICD-10-PCS Procedure Codes	CPT-4/Revenue Center Codes	Medications (generic name or HCPCS code)
Invasive Breast Cancer	174.0–174.9<br><br>All of C50*, includes: C50.011-C50.119, C50.111-C50.119, C50.211-C50.219, C50.311-C50.319, C50.411-C50.419, C50.511-C50.519, C50.811-C50.819, C50.911-C50.919			
Breast Carcinoma *in situ*	233.0<br><br>D05.10-D05.92			
Mastectomy		85.33-85.36, 85.41-85.48<br><br>0HTT0ZZ, 0HTU0ZZ, 0HTV0ZZ	19303-19307	
Breast-conserving surgery (lumpectomy, partial mastectomy)		85.20-85.23<br>0HBT0ZX, 0HBT0ZZ, 0HBT7ZX, 0HBT7ZZ, 0HBT8ZX, 0HBT8ZZ, 0HBU0ZX, 0HBU0ZZ, 0HBU7ZX, 0HBU7ZZ, 0HBU8ZX, 0HBU8ZZ, 0HBV0ZX, 0HBV0ZZ, 0HBV7ZX, 0HBV7ZZ, 0HBV8ZX, 0HBV8ZZ, 0HTWXZZ, 0HTXXZZ	19120, 19125, 19126, 19160, 19162, 19301, 19302	
Breast biopsy		85.11, 85.12<br>0HBT3ZX, 0HBT3ZZ, 0HBU3ZX, 0HBU3ZZ, 0HBV3ZX, 0HBV3ZZ, 0HBW3ZX, 0HBW3ZZ, 0HBX3ZX, 0HBX3ZZ, 0HBW0ZX, 0HBW0ZZ, 0HBW7ZX, 0HBW7ZZ, 0HBW8ZX, 0HBW8ZZ, 0HBWXZX, 0HBWXZZ, 0HBX0ZX, 0HBX0ZZ,	19081, 19082, 19083, 19084, 19085, 19086, 19100, 19101, 19102, 19103	

		0HBX7ZX, 0HBX7ZZ, 0HBX8ZX, 0HBX8ZZ, 0HBXXZX, 0HBXXZZ		
Pathology microscopic examination of tissue			88302-88388	
Chemotherapy		99.25<br><br>3E03005, 3E03305, 3E04005, 3E04305, 3E05005, 3E05305, 3E06005, 3E06305	Revenue center: 0331, 0332, 0335	96400, 96401, 96404-96440<br><br>J9000-J9999, Q0083-Q0085
Benign Breast Disease	610.0-610.3, 610.8, 610.9, 217, 238.3, 239.3, 611.72 N60.01-N60.39, N60.81-N60.99, N63, D24.1-D24.9, D48.60-D48.62, D49.3			

3. ANALYSIS VARIANTS

Table 6. List of all analysis variants according to combinations of treatment/target, risk window, outcome definition and propensity score adjustment method

Analysis ID	Treatment /Target	Comparator	Time at risk	Outcome/ Breast cancer definition	Propensity score adjustment
1	High prolactin-increasing antipsychotic	Non/low prolactin-increasing antipsychotic	Per-protocol	Nattinger Method	1:1 matching
2	High prolactin-increasing antipsychotic	Non/low prolactin-increasing antipsychotic	Intent-to-treat	Nattinger Method	1:1 matching
3	High prolactin-increasing antipsychotic	Non/low prolactin- increasing antipsychotic	Per-protocol	Rahman algorithm	1:1 matching
4	High prolactin-increasing antipsychotic	Non/low prolactin- increasing antipsychotic	Intent-to-treat	Rahman algorithm	1:1 matching
5	Moderate prolactin-increasing antipsychotic	Non/low prolactin- increasing antipsychotic	Per-protocol	Nattinger Method	1:1 matching
6	Moderate prolactin-increasing antipsychotic	Non/low prolactin- increasing antipsychotic	Intent-to-treat	Nattinger Method	1:1 matching
7	Moderate prolactin-increasing antipsychotic	Non/low prolactin- increasing antipsychotic	Per-protocol	Nattinger Method	1:1 matching
8	Moderate prolactin-increasing antipsychotic	Non/low prolactin- increasing antipsychotic	Intent-to-treat	Nattinger Method	1:1 matching
9	High prolactin-increasing antipsychotic	Non/low prolactin- increasing antipsychotic	Per-protocol	Nattinger Method	stratification
10	High prolactin-increasing antipsychotic	Non/low prolactin- increasing antipsychotic	Intent-to-treat	Nattinger Method	stratification
11	High prolactin-increasing antipsychotic	Non/low prolactin- increasing antipsychotic	Per-protocol	Rahman algorithm	stratification
12	High prolactin-increasing antipsychotic	Non/low prolactin- increasing antipsychotic	Intent-to-treat	Rahman algorithm	stratification
13	Moderate prolactin-increasing antipsychotic	Non/low prolactin- increasing antipsychotic	Per-protocol	Nattinger Method	stratification
14	Moderate prolactin-increasing antipsychotic	Non/low prolactin- increasing antipsychotic	Intent-to-treat	Nattinger Method	stratification
15	Moderate prolactin-increasing antipsychotic	Non/low prolactin- increasing antipsychotic	Per-protocol	Nattinger Method	stratification
16	Moderate prolactin-increasing antipsychotic	Non/low prolactin- increasing antipsychotic	Intent-to-treat	Nattinger Method	stratification

Table 7. Analysis variants that passed the pre-defined study diagnostics

Target	Comparator	Breast cancer outcome	PS method	Time at risk	Data
High prolactin-increasing antipsychotic users	non-prolactin-increasing antipsychotic users	Nattinger	1:1 matching	ITT	Medicaid
High prolactin-increasing antipsychotic users	non-prolactin-increasing antipsychotic users	Nattinger	Stratification	ITT	Medicaid
High prolactin-increasing antipsychotic users	non-prolactin-increasing antipsychotic users	Rahman	1:1 matching	ITT	Medicaid
High prolactin-increasing antipsychotic users	non-prolactin-increasing antipsychotic users	Rahman	Stratification	ITT	_Medicaid
High prolactin-increasing antipsychotic users	non-prolactin-increasing antipsychotic users	Rahman	Stratification	On treatment	Medicaid

PS, propensity score; ITT, intent-to-treat

4. TIME AT RISK DISTRIBUTIONS

Table 8. Time at risk in the time (days) at risk distribution expressed as minimum (min), 25th percentile (P25), median, 75th percentile (P75), and maximum (max) in the high prolactin-increasing antipsychotic (target) users and the non-prolactin-increasing antipsychotic users (comparator) cohorts after propensity score adjustment

	Min	P10	P25	Median	P75	P90	Max	PS adjustment, time at risk
**Outcome= Rahman**								
Target	2	36	112	304	994	1,376	5,208	Stratification, on-treatment
Comparator	2	38	107	289	1,003	1,315	5,205	
**Outcome= Nattinger**								
Target	2	171	451	1,176	2,983	3,562	5,298	Stratification, ITT
Comparator	2	158	423	1,008	2,834	3,446	5,299	
**Outcome= Nattinger**								
Target	2	167	436	1,115	2,886	3,426	5,298	Matching, ITT
Comparator	2	167	456	1,099	2,931	3,648	5,299	
**Outcome= Rahman**								
Target	2	171	451	1,178	2,981	3,554	2	Stratification, ITT
Comparator	2	157	422	1,007	2,833	3,445	2	
**Outcome= Rahman**								
Target	2	167	436	1,116	2,882	3,411	5,298	Matching, ITT
Comparator	2	167	454	1,095	2,931	3,647	5,299	

PS, propensity score; ITT, intent-to-treat
