# Supplementary material for: Association between prolactin increasing antipsychotic use and the risk of breast cancer: a retrospective observational cohort study in a United States Medicaid population
Source: Front Oncol. 2024 Mar 25;14:1356640. doi: 10.3389/fonc.2024.1356640 (PMC11003262; doi:10.3389/fonc.2024.1356640)
Supplement: Supplementary file 2 [file DataSheet_2.pdf]

Janssen Research & Development*

Study Protocol for Retrospective Observational Studies Using Secondary Data

Antipsychotics and breast cancer risk – a retrospective cohort study

Protocol PCSNSPA0016

* Janssen Research & Development (Janssen R&D) is a global organization that operates through different legal entities in various countries. Therefore, the legal entity acting as the sponsor for studies of Janssen R&D may vary. The term "sponsor" is used throughout the protocol to represent these various legal entities.

Status: v2.0

Date: 19 September 2022

Prepared by: Janssen Research & Development, LLC

EDMS number:

Confidentiality Statement

The information provided herein contains Company trade secrets, commercial or financial information that the Company customarily holds close and treats as confidential. The information is being provided under the assurance that the recipient will maintain the confidentiality of the information under applicable statutes, regulations, rules, protective orders or otherwise.

TABLE OF CONTENTS

1. LIST OF ABBREVIATIONS ... 4

2. RESPONSIBLE PARTIES ... 4
2.1. Investigator(s) and Authors ... 4
2.2. Sponsor ... 4

3. ABSTRACT ... 5

4. AMENDMENTS AND UPDATES ... 6

5. RATIONALE AND BACKGROUND ... 6

6. STUDY OBJECTIVES ... 7
6.1. Primary Objective(s) ... 7

7. RESEARCH METHODS ... 7
7.1. Study Design and Setting ... 7
7.2. Describe Data Source(s) ... 7
7.3. Study Population(s) ... 8
7.4. Exposure (target population 1): High prolactin-increasing antipsychotics ... 9
7.5. Exposure (target population 2): Moderate prolactin-increasing antipsychotics ... 9
7.6. Comparator: Minimal/non-prolactin-increasing antipsychotics ... 10
7.7. Exposure periods ... 10
7.8. Outcome(s) of Interest ... 10
7.3.1. Other Variables of Interest (Demographic Characteristics, Confounders, Effect Modifiers) ... 14

8. DATA ANALYSIS PLAN ... 15
8.1. Stratification by sex ... 15
8.2. Calculation of Time-at-Risk ... 15
8.3. Patient Characteristics Summary ... 16
8.4. Outcome Model Specification ... 16
8.5. Evidence Evaluation ... 16
8.6. Analysis variants ... 17
8.7. Required Diagnostic Thresholds ... 20
8.8. Power – Minimum Detectable Relative Risk (MDRR) ... 20
8.9. Attrition from propensity scores ... 20
8.10. Equipoise ... 21
8.11. Covariate Balance ... 22
8.12. Systematic error ... 22
8.13. Synthesis across databases ... 22

9. STRENGTHS AND LIMITATIONS OF THE RESEARCH METHODS ... 23

10. PROTECTION OF HUMAN SUBJECTS ... 23

11. SAFETY DATA COLLECTION AND REPORTING ... 24

12. PLANS FOR DISSEMINATING AND COMMUNICATING STUDY RESULTS ... 24

13. ANNEX (LIST OF STAND-ALONE DOCUMENTS) ... 24

14. REFERENCES ... 24

15. APPENDIX ... 26

LIST OF FIGURES

Figure 1. Study design variable assessment periods 14
Figure 2. Retrospective comparative cohort study design requiring 180-day minimum exposure period (Primary, per protocol, analysis) 16
Figure 3. Attrition within IQVIA data, female patients, high vs. low/non-prolactin increasing, ITT approach. 21
Figure 4. Preference score distribution within IQVIA data, female patients, high vs. low/non-prolactin increasing, ITT approach 21
Figure 5. Covariate balance before and after PS matching within IQVIA data, female patients, high vs. low/non-prolactin increasing, ITT approach. Each blue dot represents a different covariate. 22

LIST OF TABLES

Table 1. List of all analysis variants according to combinations of treatment/target, gender, risk window, and outcome 17
Table 2. Power for analysis within IQVIA data, female patients, high vs. low/non-prolactin increasing, ITT approach, using Rahman definition of breast cancer outcome 20

LIST OF APPENDIX TABLES

Appendix Table 1. Schizophrenia diagnosis codes 26
Appendix Table 2. Antipsychotics 28
Appendix Table 3. Codes used for Nattinger algorithm 29
Appendix Table 4. Codes used for Rahman algorithm 32
Appendix Table 5. Negative control outcomes for use in systematic error calibration 34

1. LIST OF ABBREVIATIONS

Abbreviation	Description of Abbreviated Term
CCAE	IBM® MarketScan® Commercial Database
EASE	Expected Absolute Systematic Error
ER	Emergency room
FOIA	Freedom of Information Act
ICD-9-CM	International Classification of Diseases, Ninth Revision, Clinical Modification
ICD-10-CM	International Classification of Diseases, Tenth Revision, Clinical Modification
IPTW	Inverse probability of treatment weighting
IRB	Institutional Review Board
MDCD	IBM® MarketScan® Multi-State Medicaid Database
MDCR	IBM® MarketScan® Medicare Supplemental Database
MDRR	Minimum Detectable Relative Risk
PS	Propensity score
SDM	Standardized difference of mean
SNOMED-CT	Systematized Nomenclature of Medicine -- Clinical Terms
US	United States

2. RESPONSIBLE PARTIES

2.1. Investigator(s) and Authors

Principal investigator:
David M. Kern, PhD; Epidemiology, Neuroscience and Established Products

Other investigators:
- Azza Shoaibi, PhD; Epidemiology, Observational Health and Data Analytics
- David Shearer, MD; Global Medical Safety, Medical Safety Officer
- Karl Knight, MB, BCh, MRCP; Established Products, Clinical Leader

2.2. Sponsor

Department of Epidemiology, Janssen Research & Development, LLC.

3. ABSTRACT

Background: Many retrospective observational studies have examined the relationship between use of antipsychotics and incident breast cancer. The results of these studies have been inconsistent and the methods used to conduct some of these studies are questionable, allowing for significant bias to influence the results.

Objective: To assess the association between use of prolactin increasing antipsychotics and the incidence of breast cancer.

Methods: Using multiple US-based administrative claims databases, the study will identify new users of antipsychotics which will be categorized into three groups according to their effect on prolactin levels: highly increasing, moderately increasing, and minimally/non-increasing (reference group). First use of an antipsychotic will be the index date. Incident breast cancer will be determined using two separate algorithms. The at-risk period will begin 180 days after the index date, during which time breast cancer cases will be captured. Propensity score methods will be used to control for all observed confounding, including all diagnosed comorbid conditions, all prior and concomitant medication use, demographics, and other variables found in the claims data. A Cox regression model will be used to analyze the time to incident breast cancer in each of the groups and a hazards ratio (and 95% confidence interval) will be calculated comparing they highly increasing and moderately increasing groups, separately, with the none/low increasing group. Negative control outcomes will be used to calibrate the estimates and p-values to account for residual confounding.

Strengths and Limitations: Use of multiple datasets allows for the assessment of consistency of results across different populations. Use of a per-protocol analysis and a sensitivity analysis based on an intent-to-treat design will give insight to the robustness of the results. The algorithms used for identifying breast cancer cases have a much higher validity than relying on diagnosis codes only. Propensity scores and negative controls allow for the adjustment of observed and unobserved confounding, respectively. Limiting to only patients that have a diagnosis of schizophrenia will reduce potential confounding by indication. The claims data lack clinical details such as biomarkers and tumor staging. Socioeconomic and behavioral variables are not well captured in the claims data.

4. AMENDMENTS AND UPDATES

Number	Date	Section of Study Protocol	Amendment or Update	Reason
1	19 Sep 2022	7.3, 7.7	Allow for the use of injectable therapy used for the acute management of psychotic symptoms prior to index antipsychotic use.	This study is focused on chronic use of antipsychotic and risk of breast cancer. Indexing patients on use of acute therapies (and immediately ending on-treatment follow-up) would not allow for sufficient follow up time.
1	19 Sep 2022	8.5, 8.6	Propensity score stratification added as an analysis variant.	Use of stratification (rather than matching) allows more patients to be retained in the analysis, increasing statistical power.

5. RATIONALE AND BACKGROUND

This is a follow-up to an internal white paper regarding our position on the risk of breast cancer and use of antipsychotics based on current available evidence. The development of the white paper was triggered by a recent publication in August 2021 titled "Antipsychotic use and the risk of breast cancer in women with schizophrenia: a nationwide nested case-control study in Finland" (1). The researchers concluded that their findings suggest an increased risk of breast cancer related to prolactin-increasing antipsychotics via hyperprolactinemia.

A comprehensive review of Company data and published literature, including nonclinical, clinical, epidemiology, and postmarketing safety data, was performed. The history of regulatory activities regarding health authority requests related to breast cancer and risperidone, paliperidone/paliperidone palmitate, haloperidol, and haloperidol decanoate was also reviewed. In all, a dozen retrospective observational studies examining the relationship between antipsychotic use and breast cancer were reviewed. The findings were mixed with many studies finding no association between exposure and breast cancer, while others, including Taipale et al, reported positive associations. Many of these studies suffered from limitations which put the validity of the findings in question. Such limitations include insufficient control for potential confounders, inappropriate comparators, the potential for confounding by indication, substandard statistical modeling choices, and others. Because of these limitations it was concluded that a new retrospective study should be conducted using the most appropriate epidemiologic methods.

Due to time constraints, a de novo retrospective cohort study could not be designed and executed for inclusion in the white paper. Instead, the report included descriptive statistics of breast cancer incidence in schizophrenia patients, users of antipsychotics, and the overall population. In the white paper we state "plans are in progress for a more rigorous study and data analysis", which is the rationale of this proposed study.

6. STUDY OBJECTIVES

6.1. Primary Objective(s)

To assess the risk of breast cancer in schizophrenia patients newly initiating a prolactin-increasing antipsychotic compared with those who initiate a non-prolactin-increasing antipsychotic.

7. RESEARCH METHODS

7.1. Study Design and Setting

This is a retrospective longitudinal comparative cohort study using administrative health insurance claims databases from the US. These databases include commercially insured individuals, Medicare eligible patients, and Medicaid patients.

The intake period, i.e., the period during which patients are identified and meet the inclusion criteria, will begin one year following the date of earliest available data in the database (to allow for a one-year lookback period) and for which at least one medication was approved in the treatment and comparator groups were approved and extend through the date of the most recently available data, specific to the database being used. See below for the dates of the most recently available data.

7.2. Describe Data Source(s)

Note: Feasibility will be assessed prior to analyzing the outcomes. If a data source doesn't have sufficient sample, the study report will note which databases were ultimately used in the analysis and which were not used due to lack of feasibility.

To be performed within 5 US-based administrative claims databases (Version and dates of data availability are the most recent available data at time of protocol development. Newer versions of the databases may be included if available when analysis begins.):

1. IBM® MarketScan® Commercial Database (CCAE) [v2008]: Includes data from 162 million individuals enrolled in employer-sponsored insurance health plans, during 1 Jan 2000 through 31 Jan 2022.

2. IBM® MarketScan® Multi-State Medicaid Database (MDCD) [v1978]: A claims database for 33 million Medicaid enrollees from multiple states during 1 Jan 2006 through 30 Jun 2021.

3. IBM® MarketScan® Medicare Supplemental Database (MDCR) [v2008]: Includes data for more than 10 million retirees with primary or Medicare supplemental coverage through privately insured fee-for-service, point-of-service, or capitated health plans during 1 Jan 2000 through 31 Jan 2022.

4. Optum© Clinformatics® Data Mart [v2013]: Includes 91 million members with private health insurance, who are fully insured in commercial plans or in administrative services only and Medicare Advantage (Medicare Advantage Prescription Drug coverage.

The population is representative of US commercial claims patients (0-65 years old) with some Medicare (65+ years old) during 1 May 2000 through 31 Dec 2021.

5. The IQVIA Adjudicated Health Plan Claims Data (formerly PharMetrics Plus) [v2001]: This US-based database is comprised of fully adjudicated health plan claims data and enrollment information for commercial individuals. The information is comprised of over 70 contributing health plans and self-insured employer groups throughout the United States for over more than 140 million unique enrollees during 1 Jan 2013 through 30 Nov 2021.

Data elements include outpatient pharmacy dispensing claims (coded with National Drug Codes) as well as inpatient and outpatient medical claims, which provide diagnosis codes (coded in ICD-9-CM or ICD-10-CM).

The use of the IBM MarketScan and Optum claims databases was reviewed by the New England Institutional Review Board (IRB) and was determined to be exempt from broad IRB approval, as this research project did not involve human subjects research.

Dates for patient identification will vary across data sources. Patients will be identified from one year following the date of earliest available data, to allow for a one-year lookback period, until the date of the most recently available data.

7.3. Study Population(s)

Inclusion criteria

Patients must:

1. Have a prescription fill for an antipsychotic medication listed under the 'Treatment Group' or 'Comparator Group' defined below. The earliest observed fill date will be considered the index date. *Exception*: *use of injectable therapy for the acute management of psychotic symptoms will not be counted as first antipsychotic use.*
2. Be at least 18 years of age on the index date
3. Have at least 365 days of pre-index observation in the data source.
4. Have at least one claim with a diagnosis of schizophrenia during the 365-day pre-index period, including the index date (see Appendix Table 1 for codes).
5. Have no prior fills for any antipsychotic any time prior to the index date (all time prior to indexdate-1) (see Appendix Table 2 for list of all antipsychotics). *Exception*: *use of injectable therapy for the acute management of psychotic symptoms will be allowed prior to starting chronic therapy.*
6. Not have fills for more than one antipsychotic on the index date.

Exclusion criteria

1. Diagnosis of any cancer, other than non-melanoma skin cancer, at any time prior in patient's history (up to indexdate - 1)

2. Presence of a mastectomy at any time prior in patient's history (up to indexdate - 1)
3. Diagnosis of breast cancer during the first 180 days post-index (prior to the at-risk period beginning) (indexdate to indexdate+179)
4. A prescription claim for an antipsychotic typically used for treating conditions other than schizophrenia (e.g., nausea, Tourette's syndrome) on the index date. This includes droperidol, prochlorperazine, promazine and pimozide.

7.4. Exposure (target population 1): High prolactin-increasing antipsychotics

Patients initiating a high prolactin-increasing antipsychotic used for treating schizophrenia on the index date. These antipsychotics include:

- acetophenazine
- chlorpromazine
- chlorprothixene
- fluphenazine
- haloperidol
- loxapine
- molindone
- paliperidone
- perphenazine
- risperidone
- thioridazine
- thiothixene
- trifluoperazine

The concept set of included medications can be found in Atlas here: Concept Set 3146
The cohort can be found here: https://sharedshiny-prod.jnj.com/user/grao9/epi_958/

7.5. Exposure (target population 2): Moderate prolactin-increasing antipsychotics

Patients initiating a moderate prolactin-increasing antipsychotic on the index date. These antipsychotics include:

- iloperidone
- lurasidone
- olanzapine

The concept set of included medications can be found in Atlas here: Concept Set 3610
The cohort can be found here: https://sharedshiny-prod.jnj.com/user/grao9/epi_958/

7.6. Comparator: Minimal/non-prolactin-increasing antipsychotics

Patients initiating a no/low prolactin-increasing antipsychotic on the index date. These antipsychotics include:

- aripiprazole
- asenapine
- brexpiprazole
- clozapine
- lumateperone
- quetiapine
- ziprasidone

The concept set of included medications can be found in Atlas here: Concept Set 3145
The cohort can be found here: https://sharedshiny-prod.jnj.com/user/grao9/epi_958/

7.7. Exposure periods

Exposure start date: Exposure will begin on the index date, i.e., the date of the first observed fill for non-acute antipsychotic use.

Exposure end date: Exposure will end when a patient discontinues medication (defined as a gap in treatment for any treatment within the exposure group of more than 30 days beyond the days supply of the last fill) or patient observation is censored. Censoring may occur due to any of the following conditions:

- Lost to follow-up, i.e., patient leaves the health plan and is not observed in the database
- Reaches end date of database availability
- Receives an antipsychotic for a group other than the original group (e.g., patient indexed in the "high" group fills a medication for a "moderate" or "minimal/non" antipsychotic).

Switching to an antipsychotic within the original group is allowed and will be considered as continuous use assuming the switch occurs before the criteria for discontinuation is met.

7.8. Outcome(s) of Interest

The outcome of interest is incident (newly diagnosed) breast cancer.
As part of the protocol development process, two outcome definitions will be implemented. The first is an algorithm developed by Nattinger et al (2) using linked SEER-Medicare data. The algorithm was developed using Medicare claims data and validated against the gold-standard SEER classification. The algorithm is relatively complex and may not have similar performance in other (non-Medicare) claims data. The second definition will reflect the method typically used

in breast cancer research in claims data (i.e., the “Rahman algorithm”). This algorithm is simpler, and easier to implement in our databases; however, it has not been validated against a gold-standard.

Algorithm #1: Nattinger Method

The Nattinger algorithm uses 4 steps to identify breast cancer cases. All codes used to identify each of the items can be found in Appendix Table 3. The original publication was from 2004 and did not include ICD-10-CM codes and many CPT codes that now exist. These codes have been added where relevant.

Step 1. Referred to as the “screen,” requires that a potential case have both a breast cancer diagnosis code and a breast cancer procedure code (not necessarily on the same claim) within 365 days. Only subjects satisfying this screening step are retained for further consideration. The date of the earliest observed breast cancer diagnosis is the potential event date for the outcome.

Step 2. Directly includes subjects with a high likelihood of being a case. To be classified as a case based on this step, the subject must meet both of the following criteria:

- [A mastectomy claim] or [a lumpectomy or partial mastectomy claim followed by at least one outpatient or provider claim for radiotherapy with a breast cancer diagnosis].

- At least two outpatient or provider claims on different dates, within 365 days of each other, containing breast cancer.

Subjects who pass step 2 are classified as possible incident cases and proceed to step 4. Subjects who are not classified as cases at step 2 go to step 3.

Step 3. This step of the algorithm applies to all potential cases that passed the screen (step 1) but were not directly included at step 2. In practice, this step differentiates primary breast cancer cases from women undergoing lumpectomy or partial mastectomy for benign disease or for another cancer that had metastasized to the breast. Four different variables are needed for Step 3:

- Surgery. This variable is positive (i.e., set to a value of 1) if one or more lumpectomy, partial mastectomy, or mastectomy codes are found. Otherwise, the variable is negative (set to a value of zero).

- Single Claim. This variable is positive (i.e., set to a value of 1) if a woman with lumpectomy or partial mastectomy claim had only one month in which a claim contained a primary breast cancer or a breast carcinoma-in-situ diagnosis (i.e., there do not exist two claims for breast cancer that occur between 30 and 365 days of each other). Otherwise, this variable is negative (i.e., set to 0).

- Other Cancer. This variable is positive (i.e., set to 1) if an “other cancer” code is found in one or more claims any time prior or within 30 days following the breast cancer index date. Otherwise, this variable is set to 0.

- Secondary Cancer to Breast. This variable is positive (i.e., set to 1) if a code for secondary cancer to breast is found in one or more outpatient or provider claims any time prior or within 30 days following the breast cancer index date. Otherwise, this variable is set to 0.

Once the values of the four variables have been determined, subjects can be ruled in if they have one of three combinations of the variables. These combinations are:

(1) “Surgery” = 1 and the other three variables = 0, (i.e., the patient has surgery, two breast cancer claims between 30 and 365 days of each other, no other cancer diagnosis within 365 days, and no diagnosis of secondary cancer), or

(2) “Surgery” = 1, “other cancer” = 1, and the other two variables = 0, (i.e., patient has surgery, two breast cancer claims between 30 and 365 days of each other, no diagnosis of secondary cancer, but with claims for other cancer), or

(3) “Surgery” = 1, “secondary cancer to breast” = 1, and the other two variables = 0 (i.e., patient has surgery, two breast cancer claims between 30 and 365 days of each other, no claims for other cancer, but with claims for secondary cancer to breast).

In summary, patients must have a claim for a breast cancer related surgery and at least two diagnoses between 30 and 365 days of each other and without evidence of both “other cancer” and “secondary cancer to breast”, though having one, and only one, of those is allowed. With all other combinations, the subject is declared not to be a breast cancer case (i.e., patient does not have a claim for surgery, does not have at least two claims with a breast cancer diagnosis within 30 to 365 days of each other, or has claims with diagnoses for other types of cancer and cancer secondary to breast).

Step 4. This step of the algorithm is the step to remove prevalent breast cancer cases. This step uses up to three prior years of claims of subjects classified as a case in step 2 or step 3. Such subjects are removed if they have a claim in prior years that was either positive for step 1 (the screening step) of the algorithm, or a diagnosis of prior history of breast cancer.

The cohort can be found here: https://epi.jnj.com/atlas/#/cohortdefinition/8847

Algorithm #2: Standard claims algorithm (“Rahman algorithm”)

Most typically observational studies using administrative claims data will require two claims with a diagnosis of breast cancer plus other criteria such as chemotherapy, mastectomy, or lumpectomy. A recent study published by Rahman et al (3) examined the association between

prolactin-elevating antipsychotic drugs and used an algorithm less complicated than the one detailed above, but not overly simplistic as to severely limit the validity of the classification. All codes used in the algorithm can be found in Appendix Table 4.

The algorithm is as follows:

- **Criteria 1, automatic case**: Breast cancer will be identified by the ICD-9/10-CM codes for breast cancer (invasive or *in situ*) on a claim with a CPT-4 procedure code for surgical pathology microscopic examination, indicating pathologic verification.

- **Criteria 2**: If criteria 1 is not met, the following two stipulations must be met:

    - a diagnosis of breast cancer on an inpatient facility claim or on at least 2 provider/outpatient claims separated by 30 to 180 days.

    - Evidence of surgical treatment (mastectomy or breast-conserving surgery within 1 month before through 6 months after first breast cancer diagnosis) or chemotherapy (within 6 months after first breast cancer diagnosis, chemotherapy administration coded for invasive breast cancer)

The cohort can be found here: https://epi.jnj.com/atlas/#/cohortdefinition/8863/

Figure 1. Study design variable assessment periods

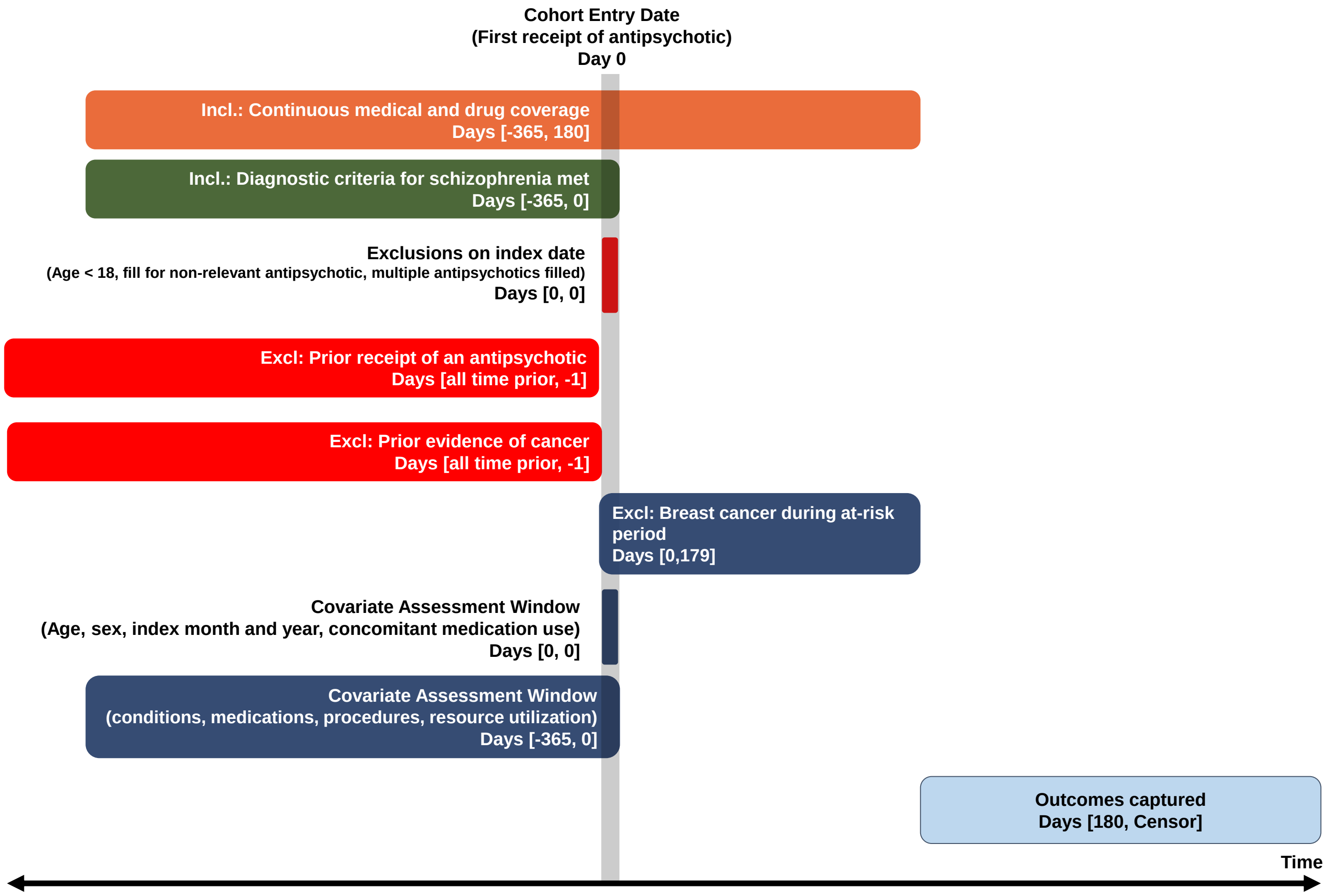

7.3.1. Other Variables of Interest (Demographic Characteristics, Confounders, Effect Modifiers)

Patient characteristics, including demographics, comorbid conditions, prior and current medication use, among others will be described for each treatment group and all will be considered for inclusion in the propensity score model unless otherwise specified. Specifically, the variables to be assessed are:

- Demographics: age (continuous and categorically), sex (used for stratification), race and ethnicity (where available)
- Time: index year, index month
- Pre-index comorbidities diagnosed during the period 30-days and 365 days prior to and including the index date
    - All conditions categorized according to the SNOMED-CT vocabulary
    - The following conditions will be specifically highlighted: bipolar disease, major depressive disorder, anxiety disorder, substance use disorders, obesity

- Prior use of medications during the 30-day and 365-day pre-index period (including the index date)
    - All drug ingredients according to the RxNorm vocabulary
    - All drug classes according to ATC classifications
    - Class level indicators: antidepressants, anxiolytics, hypnotics/sedatives
    - Drugs that may raise prolactin to be specifically highlighted: Metoclopramide, clomipramine, fenfluramine, cimetidine, or methyldopa
- Concomitant medication use (all drug ingredients with eras overlapping the index date)
- Prior procedures and measurements performed: all procedures/measurements occurring during the 365-day and 30-day pre-index periods, including the index date
- Healthcare utilization: number of outpatient/office visits, ER visits, and inpatient hospitalizations, unique prescription ingredients filled during the 365-day pre-index period (including the index date)
- Risk indices scores to be calculated using all prior patient history:
    - Charlson comorbidity index (Romano adaption)

Variables for characterization only, not included in PS model:

- Schizophrenia-related healthcare utilization: number of visits [by type] with a diagnosis of schizophrenia; any [indicator of 0 vs. 1 or more] inpatient hospitalization with a primary diagnosis of schizophrenia]

8. DATA ANALYSIS PLAN

8.1. Stratification by sex

All analyses will be stratified by sex, separately for males and females. There will be no analysis which combines men and women into a single group.

8.2. Calculation of Time-at-Risk

At-risk period start date: The at-risk period will start **6 months (180 days)** following the index date. The six-month lag is required to make a reasonable inference that the exposure contributed to the incidence of cancer. While it is likely that a tumor may have been growing for years or decades prior to the first diagnosis, it is not feasible to require such extended observation periods. The hypothesized effect of prolactin on breast cancer is increased tumor growth not the formation of a tumor or initial cell mutation, thus long lag periods to detect *new* tumor formation are not necessary.

At-risk period end date: Two different analyses will be conducted, based on differing end date definitions

- Primary analysis - Per protocol analysis: The at-risk period will end at the end of exposure or presence of a censoring event
- Sensitivity analysis - Intent to treat: The at-risk period will end at the end of observation in the database, regardless of continuous exposure to the index treatment group or exposure to the comparator. This analysis will not impose a minimum length of exposure to drug, but the at-risk period will begin on day 180.

Figure 2. Retrospective comparative cohort study design requiring 180-day minimum exposure period (Primary, per protocol, analysis)

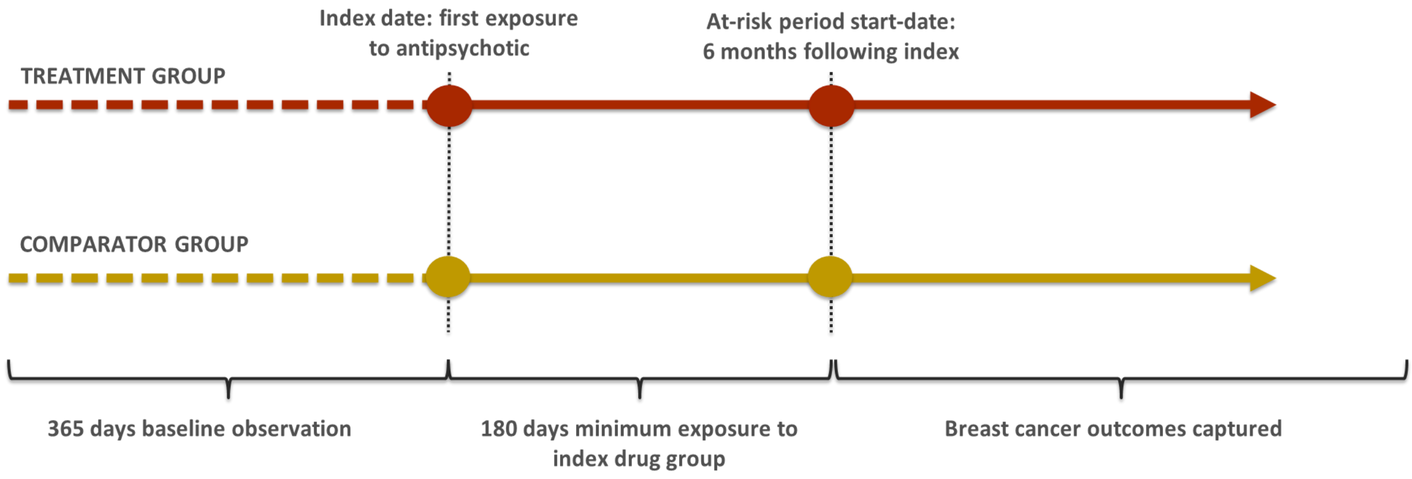

8.3. Patient Characteristics Summary

Means and proportions will be reported for all patient characteristic variables found in Section 1818167096.1574092943.7.3.1. Absolute standardized differences will be reported comparing the treatment cohort to the comparator cohort. Any differences ≤0.10 will be considered “balanced”.

8.4. Outcome Model Specification

A Cox proportional hazards model conditioned on the propensity score matching strata will be performed. Calibrated and uncalibrated hazard ratios, 95% confidence intervals, and p-values will be reported. Only calibrated hazard ratios will be used for inference. All analyses will be stratified by sex.

8.5. Evidence Evaluation

Propensity score matching and stratification techniques will be used to control for observed potential confounding. The variables described in Section 1818167096.1574092943.7.3.1 will be considered for inclusion in the propensity score model used to predict treatment arm. A regression model using L1 regularization (LASSO regression) will be used to select the most relevant covariates for inclusion in the propensity score model. Separate propensity score models will be developed for males and females.

We will evaluate the preference score distributions from the propensity score model and determine the proportion of patients in equipoise between (a preference score between 0.30 and 0.70). If the overlap of preference scores is sufficient to support justify matching the populations (proportion in equipoise >~50%) we will continue with the process. For matching, propensity scores will be used to match the cohorts (1-to-1 matching with a caliper of 0.2 of the standardized logit score), in which a subset of the cohorts which are most similar to each other will be retained. For stratification, to control for measured confounders, we will allocate target and comparator patients into five strata defined by the distribution of the propensity score. Effects will be estimated within strata (each of which contains patients who resemble each other by nature of having similar propensity scores) then combined into a weighted average. Propensity score stratification using 5 strata has been demonstrated to control the bias produced by measured confounders.

Covariate balance between the cohorts will be assessed before and after the PS matching is conducted. Covariates will be considered well balanced between cohorts if the standardized mean difference is ≤0.10. If any covariates remain unbalanced between cohorts the propensity score model may be adjusted and balance reassessed.

If sufficient overlap between propensity scores isn't present and/or if matching/stratification the cohorts does not result in groups that are well balanced on nearly all patient characteristics (including, but not limited to those in Section 1818167096.1574092943.7.3.1), a decision will be made to not proceed with the study due to an inability to make unbiased inferences regarding the relationship between exposure and outcome.

To adjust for potential unobserved confounding and residual bias, empirical calibration using negative controls will be performed. Both uncalibrated and calibrated results will be reported, but only calibrated results will be used for inference.

A sample of the analysis diagnostics are found in the sections below. This includes diagnostics for the comparison of high prolactin-increasing vs. none/low, within females, using the per-protocol analysis, from the IQVIA database. This analysis will be conducted for all analysis variants (see Section 8.6) within each of the databases.

8.6. Analysis variants

Due to the different population strata (males, females), risk window end dates (end of observation, end of drug use), outcome/breast cancer definition (Nattinger, Rahman), and treatment/target groups (high prolactin-increasing, moderate prolactin-increasing), there are 16 unique analysis variants.

Table 1. List of all analysis variants according to combinations of treatment/target, gender, risk window, and outcome

Analysis ID	Treatment /Target	Comparator	Gender	Risk window end	Outcome/ Breast cancer definition	Propensity score adjustment

1	High prolactin-increasing antipsychotic	Minimal/non-prolactin- increasing antipsychotic	Female	End of observation	Nattinger Method	1:1 matching
2	High prolactin-increasing antipsychotic	Minimal/non-prolactin- increasing antipsychotic	Male	End of observation	Nattinger Method	1:1 matching
3	High prolactin-increasing antipsychotic	Minimal/non-prolactin- increasing antipsychotic	Female	End of drug use	Nattinger Method	1:1 matching
4	High prolactin-increasing antipsychotic	Minimal/non-prolactin- increasing antipsychotic	Male	End of drug use	Nattinger Method	1:1 matching
5	High prolactin-increasing antipsychotic	Minimal/non-prolactin- increasing antipsychotic	Female	End of observation	Rahman algorithm	1:1 matching
6	High prolactin-increasing antipsychotic	Minimal/non-prolactin- increasing antipsychotic	Male	End of observation	Rahman algorithm	1:1 matching
7	High prolactin-increasing antipsychotic	Minimal/non-prolactin- increasing antipsychotic	Female	End of drug use	Rahman algorithm	1:1 matching
8	High prolactin-increasing antipsychotic	Minimal/non-prolactin- increasing antipsychotic	Male	End of drug use	Rahman algorithm	1:1 matching
9	Moderate prolactin-increasing antipsychotic	Minimal/non-prolactin- increasing antipsychotic	Female	End of observation	Nattinger Method	1:1 matching
10	Moderate prolactin-increasing antipsychotic	Minimal/non-prolactin- increasing antipsychotic	Male	End of observation	Nattinger Method	1:1 matching
11	Moderate prolactin-increasing antipsychotic	Minimal/non-prolactin- increasing antipsychotic	Female	End of drug use	Nattinger Method	1:1 matching
12	Moderate prolactin-increasing antipsychotic	Minimal/non-prolactin- increasing antipsychotic	Male	End of drug use	Nattinger Method	1:1 matching
13	Moderate prolactin-increasing antipsychotic	Minimal/non-prolactin- increasing antipsychotic	Female	End of observation	Nattinger Method	1:1 matching
14	Moderate prolactin-increasing antipsychotic	Minimal/non-prolactin- increasing antipsychotic	Male	End of observation	Nattinger Method	1:1 matching
15	Moderate prolactin-increasing antipsychotic	Minimal/non-prolactin- increasing antipsychotic	Female	End of drug use	Nattinger Method	1:1 matching
16	Moderate prolactin-increasing antipsychotic	Minimal/non-prolactin- increasing antipsychotic	Male	End of drug use	Nattinger Method	1:1 matching
17	High prolactin-increasing antipsychotic	Minimal/non-prolactin- increasing antipsychotic	Female	End of observation	Nattinger Method	stratification

18	High prolactin-increasing antipsychotic	Minimal/non-prolactin- increasing antipsychotic	Male	End of observation	Nattinger Method	stratification
19	High prolactin-increasing antipsychotic	Minimal/non-prolactin- increasing antipsychotic	Female	End of drug use	Nattinger Method	stratification
20	High prolactin-increasing antipsychotic	Minimal/non-prolactin- increasing antipsychotic	Male	End of drug use	Nattinger Method	stratification
21	High prolactin-increasing antipsychotic	Minimal/non-prolactin- increasing antipsychotic	Female	End of observation	Rahman algorithm	stratification
22	High prolactin-increasing antipsychotic	Minimal/non-prolactin- increasing antipsychotic	Male	End of observation	Rahman algorithm	stratification
23	High prolactin-increasing antipsychotic	Minimal/non-prolactin- increasing antipsychotic	Female	End of drug use	Rahman algorithm	stratification
24	High prolactin-increasing antipsychotic	Minimal/non-prolactin- increasing antipsychotic	Male	End of drug use	Rahman algorithm	stratification
25	Moderate prolactin-increasing antipsychotic	Minimal/non-prolactin- increasing antipsychotic	Female	End of observation	Nattinger Method	stratification
26	Moderate prolactin-increasing antipsychotic	Minimal/non-prolactin- increasing antipsychotic	Male	End of observation	Nattinger Method	stratification
27	Moderate prolactin-increasing antipsychotic	Minimal/non-prolactin- increasing antipsychotic	Female	End of drug use	Nattinger Method	stratification
28	Moderate prolactin-increasing antipsychotic	Minimal/non-prolactin- increasing antipsychotic	Male	End of drug use	Nattinger Method	stratification
29	Moderate prolactin-increasing antipsychotic	Minimal/non-prolactin- increasing antipsychotic	Female	End of observation	Nattinger Method	stratification
30	Moderate prolactin-increasing antipsychotic	Minimal/non-prolactin- increasing antipsychotic	Male	End of observation	Nattinger Method	stratification
31	Moderate prolactin-increasing antipsychotic	Minimal/non-prolactin- increasing antipsychotic	Female	End of drug use	Nattinger Method	stratification
32	Moderate prolactin-increasing antipsychotic	Minimal/non-prolactin- increasing antipsychotic	Male	End of drug use	Nattinger Method	stratification

8.7. Required Diagnostic Thresholds

The study is subject to a set of pre-determined standardized diagnostics that must pass prior to results review. Each diagnostic has a diagnostic failure threshold, and failure of any diagnostic will result in that analysis not being unblinded, i.e., results will not be reported.

8.8. Power – Minimum Detectable Relative Risk (MDRR)

The MDRR for a given observed sample size (after propensity score matching) will be computed using an α=0.05, β=0.20. The diagnostic failure threshold is an MDRR value of greater than or equal to 10.

Table 2. Power for analysis within IQVIA data, female patients, high vs. low/non-prolactin increasing, ITT approach, using Rahman definition of breast cancer outcome

Target subjects	Comparator subjects	Target years	Comparator years	Comparator events	Comparator IR (per 1,000 PY)	MDRR
1,191	1,191	1,944	1,900	<5	<2.63	>Inf

Due to lack of sufficient number of events the MDRR is >10 and there is insufficient power to detect a difference.

8.9. Attrition from propensity scores

From all individuals in the exposure cohort, the number of persons in the final analysis after all attrition due to propensity score adjustments will be assessed. Patients may be removed from the analysis if they are part of the comparator or did not have a match. The diagnostic failure threshold is an attrition greater than 50% due to lack of a match.

Figure 3. Attrition within IQVIA data, female patients, high vs. low/non-prolactin increasing, ITT approach.

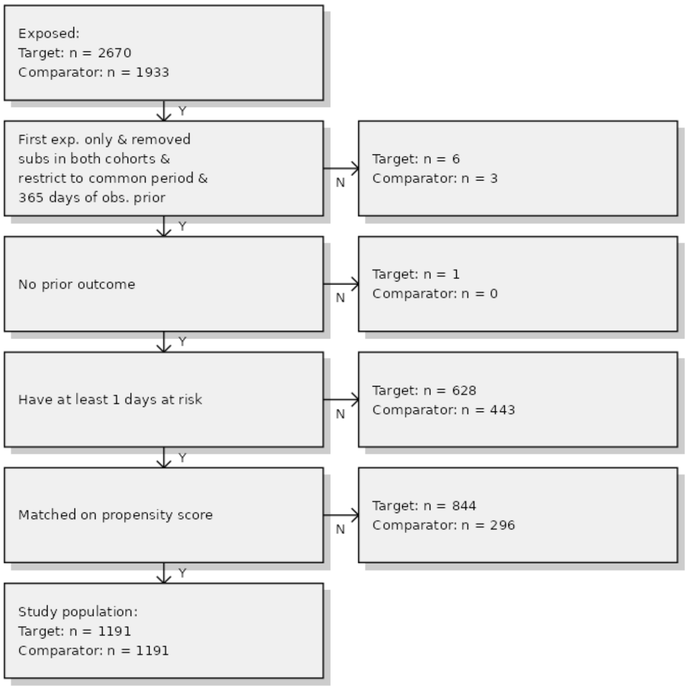

8.10. Equipoise

The proportion of the population with a preference score (4) between 0.3 and 0.7, called equipoise, will be computed. The preference score is a linear transformation of the propensity score. The diagnostic failure threshold is an equipoise value of less than 10%.

Figure 4. Preference score distribution within IQVIA data, female patients, high vs. low/non-prolactin increasing, ITT approach

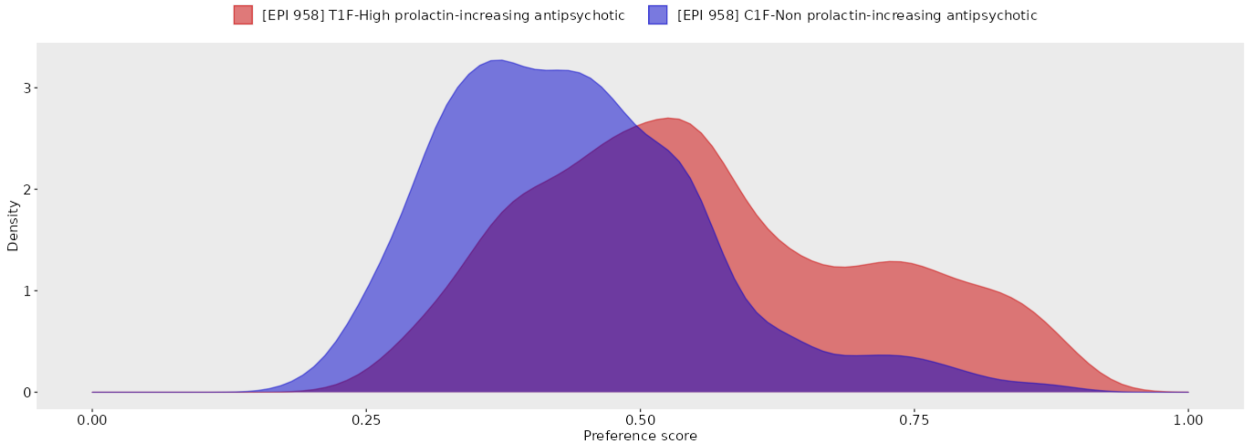

The high overlap indicates subjects in the two groups were similar in terms of their predicted probability of receiving one treatment over the other.

8.11. Covariate Balance

The standardized difference of mean (SDM) (5) will be computed for every covariate that was used to balance the two cohorts. Covariates will be considered well balanced if the SDM is less than 0.10.

Figure 5. Covariate balance before and after PS matching within IQVIA data, female patients, high vs. low/non-prolactin increasing, ITT approach. Each blue dot represents a different covariate.

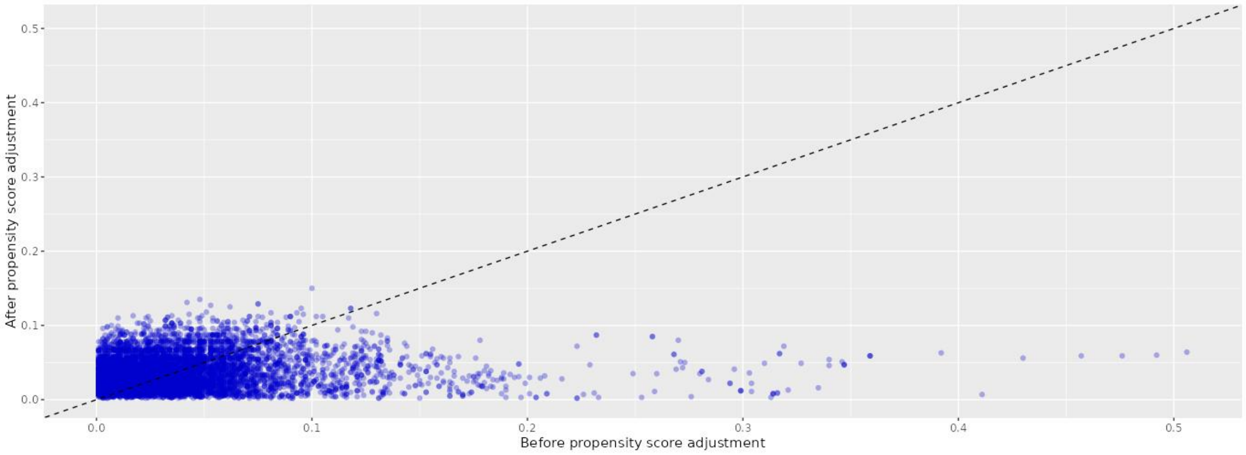

While most covariates fall <0.10 SDM after matching a number of covariates are above this threshold. Further investigation of covariates that are unbalanced is warranted to understand possible impact on confounding, such as their prevalence and observed associations with the outcomes, and whether differences in prevalence of these covariates could bias results.

8.12. Systematic error

A systematic error distribution using negative control estimates (6) will be fit and will be summarized as the Expected Absolute Systematic Error (EASE). An EASE of 0 means all variations in the negative control estimates can be explained by random error (as expressed for example in the CI). We expect at least 80% of the negative controls to fall within the null. Statistical corrections will be applied to the outcome estimate, confidence interval, and p-values according to the distribution of the observed effects within the negative control outcomes.

The list of all negative control outcomes is found in Appendix Table 5.

8.13. Synthesis across databases

For evaluation, we will combine effect-size estimates across databases using Bayesian random-effects meta-analysis using non-normal likelihood approximation to avoid bias due to small counts. Empirical calibration will be performed by first computing meta-analytic estimates for all negative controls (7), use those to fit empirical null distributions, and finally calibrated the meta-analytic estimates for the outcomes of interest.

9. STRENGTHS AND LIMITATIONS OF THE RESEARCH METHODS

Strengths:

- The use of multiple databases allows for the opportunity to assess the consistency of observed effects across various populations of the US, including those with and without commercial insurance.
- Cohort studies allow direct estimation of incidence rates following exposure of interest
- The new-user design can capture events following treatment exposures while avoiding confounding from previous treatment effects (prevalent user bias). New use allows for a clear exposure index date designation.
- Propensity score adjustment allows balancing on a large number of baseline potential confounders.
- The use of a set of negative control outcomes allows for estimation of residual bias inherent to the study design and data and the ability to calibrate our study results to account for this residual bias.

Limitations:

- The outcome of breast cancer relies on algorithms using administrative claims data, which, while shown to have high validity, are not perfect in terms of sensitivity or specificity.
- The presence of a diagnosis code signals a stage at which a tumor growth has led to seeking and receiving healthcare to address the cancer. Initial cell mutations and early tumor growth may have begun years prior to a diagnosis code being observed. However, in vitro and in vivo studies support that prolactin is involved in processes related to late-stage carcinogenic effects of breast cancer, including increasing cell proliferation and reducing apoptosis (8). Thus, prolactin levels may only be important after a preclinical lesion has developed.
- The claims data do not have information on biomarkers, tumor staging, or other clinical measures for the outcome.
- Causality between drug exposure and any given event cannot be drawn for individual cases.
- Socioeconomic variables (such as race/ethnicity, education, income), behavioral variables (such as diet, exercise, tobacco and other drug use) are not available or may not be completely captured from these databases.
- Potential residual confounding may also occur due to incomplete capture of other breast cancer risk factors (genetic mutations, family hx, breast density, parity, menopause status etc.)
- Adjustment by propensity score may not completely remove confounding bias

10. PROTECTION OF HUMAN SUBJECTS

The New England Institutional Review Board (IRB) has determined that studies conducted in IBM MarketScan and Optum Extended databases are exempt from study-specific IRB review, as these studies do not qualify as human subjects research

11. SAFETY DATA COLLECTION AND REPORTING

This study uses coded data that already exist in an electronic database. In this type of database, the minimum criteria for reporting an adverse event (i.e., identifiable patient, identifiable reporter, a suspect product, and event) are not available, and adverse events are not reportable as individual case safety reports [EMA GVP 2017]. The study results will be assessed for medically important results.

12. PLANS FOR DISSEMINATING AND COMMUNICATING STUDY RESULTS

Results of this study will be submitted for publication in a peer reviewed journal.

13. ANNEX (LIST OF STAND-ALONE DOCUMENTS)

Document Number	Date	Title
1		
2		
...		

14. REFERENCES

1. Taipale H, Gomm W, Broich K, Maier W, Tolppanen AM, Tanskanen A, et al. Use of Antiepileptic Drugs and Dementia Risk-an Analysis of Finnish Health Register and German Health Insurance Data. J Am Geriatr Soc. 2018/03/23. 2018;66(6):1123–9.

2. Nattinger AB, Laud PW, Bajorunaite R, Sparapani RA, Freeman JL. An algorithm for the use of Medicare claims data to identify women with incident breast cancer. Health Serv Res. 2004 Dec;39(6 Pt 1):1733–49.

3. Rahman T, Sahrmann JM, Olsen MA, Nickel KB, Miller JP, Ma C, et al. Risk of Breast Cancer With Prolactin Elevating Antipsychotic Drugs: An Observational Study of US Women (Ages 18-64 Years). J Clin Psychopharmacol. 2022;42(1):7–16.

4. Walker A, Patrick A, Lauer M, Hornbrook M, Marin M, Platt R, Roger V, Stang P SS. A tool for assessing the feasibility of comparative effectiveness research. Comp Eff Res. 2013;3:11–20.

5. Austin PC. Assessing balance in measured baseline covariates when using many-to-one matching on the propensity-score. Pharmacoepidemiol Drug Saf. 2008 Dec;17(12):1218–25.

6. Schuemie MJ, Hripcsak G, Ryan PB, Madigan D, Suchard MA. Empirical confidence interval calibration for population-level effect estimation studies in observational healthcare data. Proc Natl Acad Sci U S A. 2018 Mar;115(11):2571–7.

7. Schuemie MJ, Chen Y, Madigan D, Suchard MA. Combining cox regressions across a heterogeneous distributed research network facing small and zero counts. Stat Methods Med Res [Internet]. 2021 Nov 29;31(3):438–50. Available from: https://doi.org/10.1177/09622802211060518

8. Peuskens J, Pani L, Detraux J, De Hert M. The effects of novel and newly approved antipsychotics on serum prolactin levels: a comprehensive review. CNS Drugs. 2014 May;28(5):421–53.

15. APPENDIX

Appendix Table 1. Schizophrenia diagnosis codes

Code	Name	Vocabulary
F20.0	Paranoid schizophrenia	ICD10CM
F20.1	Disorganized schizophrenia	ICD10CM
F20.2	Catatonic schizophrenia	ICD10CM
F20.3	Undifferentiated schizophrenia	ICD10CM
F20.5	Residual schizophrenia	ICD10CM
F20.89	Other schizophrenia	ICD10CM
F20.9	Schizophrenia, unspecified	ICD10CM
295.00	Simple type schizophrenia, unspecified	ICD9CM
295.01	Simple type schizophrenia, subchronic	ICD9CM
295.02	Simple type schizophrenia, chronic	ICD9CM
295.03	Simple type schizophrenia, subchronic with acute exacerbation	ICD9CM
295.04	Simple type schizophrenia, chronic with acute exacerbation	ICD9CM
295.05	Simple type schizophrenia, in remission	ICD9CM
295.10	Disorganized type schizophrenia, unspecified	ICD9CM
295.11	Disorganized type schizophrenia, subchronic	ICD9CM
295.12	Disorganized type schizophrenia, chronic	ICD9CM
295.13	Disorganized type schizophrenia, subchronic with acute exacerbation	ICD9CM
295.14	Disorganized type schizophrenia, chronic with acute exacerbation	ICD9CM
295.15	Disorganized type schizophrenia, in remission	ICD9CM
295.20	Catatonic type schizophrenia, unspecified	ICD9CM
295.21	Catatonic type schizophrenia, subchronic	ICD9CM
295.22	Catatonic type schizophrenia, chronic	ICD9CM
295.23	Catatonic type schizophrenia, subchronic with acute exacerbation	ICD9CM
295.24	Catatonic type schizophrenia, chronic with acute exacerbation	ICD9CM
295.25	Catatonic type schizophrenia, in remission	ICD9CM
295.30	Paranoid type schizophrenia, unspecified	ICD9CM
295.31	Paranoid type schizophrenia, subchronic	ICD9CM
295.32	Paranoid type schizophrenia, chronic	ICD9CM
295.33	Paranoid type schizophrenia, subchronic with acute exacerbation	ICD9CM
295.34	Paranoid type schizophrenia, chronic with acute exacerbation	ICD9CM
295.35	Paranoid type schizophrenia, in remission	ICD9CM
295.50	Latent schizophrenia, unspecified	ICD9CM
295.51	Latent schizophrenia, subchronic	ICD9CM
295.52	Latent schizophrenia, chronic	ICD9CM
295.53	Latent schizophrenia, subchronic with acute exacerbation	ICD9CM
295.54	Latent schizophrenia, chronic with acute exacerbation	ICD9CM
295.55	Latent schizophrenia, in remission	ICD9CM
295.60	Schizophrenic disorders, residual type, unspecified	ICD9CM
295.61	Schizophrenic disorders, residual type, subchronic	ICD9CM
295.62	Schizophrenic disorders, residual type, chronic	ICD9CM

295.63	Schizophrenic disorders, residual type, subchronic with acute exacerbation	ICD9CM
295.64	Schizophrenic disorders, residual type, chronic with acute exacerbation	ICD9CM
295.65	Schizophrenic disorders, residual type, in remission	ICD9CM
295.80	Other specified types of schizophrenia, unspecified	ICD9CM
295.81	Other specified types of schizophrenia, subchronic	ICD9CM
295.82	Other specified types of schizophrenia, chronic	ICD9CM
295.83	Other specified types of schizophrenia, subchronic with acute exacerbation	ICD9CM
295.84	Other specified types of schizophrenia, chronic with acute exacerbation	ICD9CM
295.85	Other specified types of schizophrenia, in remission	ICD9CM
295.90	Unspecified schizophrenia, unspecified	ICD9CM
295.91	Unspecified schizophrenia, subchronic	ICD9CM
295.92	Unspecified schizophrenia, chronic	ICD9CM
295.93	Unspecified schizophrenia, subchronic with acute exacerbation	ICD9CM
295.94	Unspecified schizophrenia, chronic with acute exacerbation	ICD9CM
295.95	Unspecified schizophrenia, in remission	ICD9CM

Appendix Table 2. Antipsychotics

Antipsychotic	High/Moderate/Low/Other
aripiprazole	L
asenapine	L
brexpiprazole	L
clozapine	L
lumateperone	L
quetiapine	L
ziprasidone	L
iloperidone	M
lurasidone	M
olanzapine	M
acetophenazine	T
chlorpromazine	T
chlorprothixene	T
fluphenazine	T
haloperidol	T
loxapine	T
molindone	T
paliperidone	T
perphenazine	T
risperidone	T
thioridazine	T
thiothixene	T
trifluoperazine	T
droperidol	O
pimozide	O
prochlorperazine	O
promazine	O

"Other" antipsychotics are used to establish clean period of no prior antipsychotic use but are not used for any of the exposure groups

The Atlas concept set of all antipsychotics can be found here:
https://epi.jnj.com/atlas/#/conceptset/1127/expression

Appendix Table 3. Codes used for Nattinger algorithm

Diagnosis or Procedure	Diagnosis codes (ICD-9-CM/ ICD-10-CM)	Procedure codes (CPT/HCPCS and ICD-9 Procedure, ICD-10 PCS)
Breast cancer **	174.0–174.9, 175.0-175.9<br>All of C50*, includes:<br>C50.011-C50.119, C50.111-C50.119, C50.211-C50.219, C50.311-C50.319, C50.411-C50.419, C50.511-C50.519, c50.6-c50.629	
Carcinoma-in-situ (breast) **	233.0<br>D05.10-D05.92	
Mastectomy †,§		ICD9: 85.33-85.36, 85.41-85.48<br>ICD10-CM: 0HTT0ZZ, 0HTU0ZZ, 0HTV0ZZ<br>CPT: 19180, 19182, 19200, 19220, 19240, 19303-19307
Lumpectomy and partial mastectomy †,§		ICD9: 85.20-85.23<br>ICD10-CM, 0HBT0ZZ, 0HBT7ZZ, 0HBT8ZZ, 0HBU0ZZ, 0HBU7ZZ, 0HBU8ZZ, 0HBV0ZZ, 0HBV7ZZ, 0HBV8ZZ, 0HTWXZZ, 0HTXXZZ, 0HBT3ZZ, 0HBU3ZZ, 0HBV3ZZ, 0HBW3ZZ, 0HBX3ZZ, 0HBW0ZZ, 0HBW7ZZ, 0HBW8ZZ, 0HBWXZZ, 0HBX0ZZ, 0HBX7ZZ, 0HBX8ZZ, 0HBXXZZ<br>CPT: 19120, 19125, 19126, 19160, 19162, 19301, 19302

Biopsy †		ICD9: 85.11, 85.12<br>ICD10-CM: 0HBT3ZX, , 0HBU3ZX, , 0HBV3ZX, , 0HBW3ZX, , 0HBX3ZX, , 0HBW0ZX, , 0HBW7ZX, 0HBW8ZX, , 0HBWXZX, , 0HBX0ZX, , 0HBX7ZX, 0HBX8ZX, , 0HBXXZX, , 0HBX8ZX, 0HBX7ZX, 0HBX3ZX,<br>0HBX0ZX, 0HBWXZX, 0HBW8ZX, 0HBW7ZX, 0HBW3ZX, 0HBW0ZX<br>CPT: 19081, 19082, 19083, 19084, 19085, 19086, 19100, 19101, 19102, 19103, 19125, 19126, 77031
Lymph node dissection associated with breast cancer (thorax, axillary, mammary) †,§		ICD9: 40.22, 40.23, 40.3, 40.51<br>ICD10-CM: 07B5*, 07B6*, 07B7*, 07B8*, 07B9*,07T50ZZ, 07T54ZZ, 07T60ZZ, 07T64ZZ<br><br>CPT: 38525, 38530, 38542, 38740, 38745, 38746
Secondary cancer to breast ^	198.2, 198.81, C79.2, C79.81	
Other cancer ^	140.0-208.91 (excluding 174.0-175.9, 196.0-196.9, 198.2, 198.81, 199.2), 230-239.9 (excluding 233.0, 238.3, 239.3)<br>C00-C80 (excluding C50.*, C77.*, C79.2, C79.81), D00-D09 (excluding D05.10-D05.92), D37-D48 (excluding D48.60, D48.61, D48.62,), D49 (excluding D49.3)	
History of breast cancer #	V10.3, Z85.3	
Tumor in breast of uncertain nature	238.3, 239.3<br><br>D48.60, D48.61, D48.62, D49.3	

Radiation therapy +	92.20–92.29, 92.30-92.39, 92.41	ICD9-CM procedure codes: 92.20–92.29, 92.30-92.39, 92.41<br>ICD10 PCS codes: DM0*, DM1*, DM2*, DMY*<br>CPT/HCPCS codes:<br>77371-77387, G6001, G6002, G6015-G6017 (stereoactic radiation),<br>77399-77417, G6003 - G6014 (radiation treatment),<br>77423-77425 (neutron beam treatment),<br>77427-77499 (radiation treatment management),<br>77520-77525 (proton beam radiation),<br>77600-77615 (radiation hyperthermia treatment),<br>77620 (clinical intracitary radiation hyperthermia treatment),<br>77750-77799 (clinical brachytherapy radiation treatment)<br>G6003 - G6014

** Diagnoses for Step 1
† Procedures for Step 1
§ Procedures for ‘‘Surgery’’ variable in Step 3
^ other variables used in Step 3
# Variable used in Step 4
+ Variable used in Step 2

Appendix Table 4. Codes used for Rahman algorithm

Category	ICD-9-CM/ICD-10 Diagnosis Codes	ICD-9-CM/ICD-10-PCS Procedure Codes	CPT-4/Revenue Center Codes	Medications (generic name or HCPCS code)
Invasive Breast Cancer	174.0–174.9<br><br>All of C50*, includes: C50.011-C50.119, C50.111-C50.119, C50.211-C50.219, C50.311-C50.319, C50.411-C50.419, C50.511-C50.519, C50.811-C50.819, C50.911-C50.919			
Breast Carcinoma *in situ*	233.0<br>D05.10-D05.92			
Mastectomy		85.33-85.36, 85.41-85.48<br><br>0HTT0ZZ, 0HTU0ZZ, 0HTV0ZZ	19303-19307	
Breast-conserving surgery (lumpectomy, partial mastectomy)		85.20-85.23<br>0HBT0ZX, 0HBT0ZZ, 0HBT7ZX, 0HBT7ZZ, 0HBT8ZX, 0HBT8ZZ, 0HBU0ZX, 0HBU0ZZ, 0HBU7ZX, 0HBU7ZZ, 0HBU8ZX, 0HBU8ZZ, 0HBV0ZX, 0HBV0ZZ, 0HBV7ZX, 0HBV7ZZ, 0HBV8ZX, 0HBV8ZZ, 0HTWXZZ, 0HTXXZZ	19120, 19125, 19126, 19160, 19162, 19301, 19302	
Breast biopsy		85.11, 85.12<br>0HBT3ZX, 0HBT3ZZ, 0HBU3ZX, 0HBU3ZZ, 0HBV3ZX, 0HBV3ZZ, 0HBW3ZX, 0HBW3ZZ,	19081, 19082, 19083, 19084, 19085, 19086, 19100, 19101, 19102, 19103	

		0HBX3ZX, 0HBX3ZZ, 0HBW0ZX, 0HBW0ZZ, 0HBW7ZX, 0HBW7ZZ, 0HBW8ZX, 0HBW8ZZ, 0HBWXZX, 0HBWXZZ, 0HBX0ZX, 0HBX0ZZ, 0HBX7ZX, 0HBX7ZZ, 0HBX8ZX, 0HBX8ZZ, 0HBXXZX, 0HBXXZZ		
Pathology microscopic examination of tissue			88302-88388	
Chemotherapy		**99.25**<br><br>**3E03005, 3E03305, 3E04005, 3E04305, 3E05005, 3E05305, 3E06005, 3E06305**	Revenue center: 0331, 0332, 0335	96400, 96401, 96404-96440<br><br>J9000-J9999, Q0083-Q0085
Benign Breast Disease	610.0-610.3, 610.8, 610.9, 217, 238.3, 239.3, 611.72<br>N60.01-N60.39, N60.81-N60.99, N63, D24.1-D24.9, D48.60-D48.62, D49.3			

Appendix Table 5. Negative control outcomes for use in systematic error calibration

conceptId	conceptName
374375	Impacted cerumen
440276	Infection AND/OR inflammatory reaction due to internal prosthetic device, implant AND/OR graft
257683	Posterior rhinorrhea
440320	Infection due to Escherichia coli
440321	Helicobacter-associated disease
74052	Labyrinthitis
195590	Urethral stricture
436339	Bacterial infection due to Klebsiella pneumoniae
440638	Lyme disease
79833	Ménière's disease
438064	Bacterial infection due to Pseudomonas
195862	Urethritis
375292	Perforation of tympanic membrane
193520	Urinary bladder stone
432508	Mechanical complication of nervous system device, implant AND/OR graft
194683	Obstructed umbilical hernia
72415	Synovial plica
141216	Molluscum contagiosum infection
436957	Functional disorders of polymorphonuclear neutrophils
4025663	Obstructed inguinal hernia
438350	Pneumocystosis
4241530	Asymptomatic human immunodeficiency virus infection
435228	Common variable agammaglobulinemia
436621	Meningococcal infectious disease
133327	Viremia
80008	Breakage of joint prosthesis
4265485	Bacteriuria
196733	Urethral syndrome
74723	Arthropathy associated with a neurological disorder
436900	Mycoplasma infection
439025	Dacryocystitis
372925	Cholesteatoma
4012934	Homocystinuria
4100858	Selective immunoglobulin G deficiency
433171	Selective immunoglobulin A deficiency
433405	Proteus infection
439035	Otosclerosis
437217	Coccidioidomycosis
378765	Tympanosclerosis
74124	Traumatic arthropathy
435186	Disease due to Rhinovirus
194996	Postoperative urethral stricture
441235	Large cell anaplastic lymphoma
4057087	Obstructed femoral hernia

439730	Babesiosis
79732	Genitourinary chlamydia infection
437202	Giardiasis
440642	Cat scratch disease
200774	Detrusor and sphincter dyssynergia
4205455	Disseminated herpes zoster
381585	Cholesteatoma of middle ear
443204	Human ehrlichiosis
440035	Cryptococcosis
440653	Toxoplasmosis
434893	Selective immunoglobulin M deficiency
40485064	Latent syphilis
440330	Bartonellosis
4215809	Hypercalciuria
435514	Lymphoproliferative disorder following transplantation
436897	Pasteurella infection
4292547	Lipomatosis
79104	Loose body in joint
434567	Q fever
434575	Spotted fevers
432873	Hereditary fructosuria
438067	Malaria
432830	Zygomycosis
432287	Hyperimmunoglobulin M syndrome
4089968	Rocky Mountain spotted fever
441487	Frostbite
381867	Chronic enlargement of lacrimal gland
439788	Galactosemia
438365	Phaeohyphomycosis
432829	Yaws
78238	Rupture of synovium
4149583	Cryoglobulinemia
433135	Infection by Taenia
443739	Disease due to Rickettsia
4273323	Rubella
436040	Infection by Strongyloides
4097550	Legionella infection
436906	Disease caused by rickettsiae
4062405	Rhinophyma
432246	Leptospirosis
437787	Secondary syphilis
436630	Visceral herpes simplex
4312704	Necrobiosis lipoidica
434859	Sporotrichosis
4003404	Inactive tuberculosis
4104668	Nocardiosis

432821	Leprosy
433777	Dacryolith
440043	Echinococcosis
